# Suppression of PFKFB3-driven glycolysis restrains endothelial-to-mesenchymal transition and fibrotic response

**DOI:** 10.1038/s41392-022-01097-6

**Published:** 2022-09-01

**Authors:** Hao Zeng, Ting Pan, Meiling Zhan, Renaguli Hailiwu, Baolin Liu, Hua Yang, Ping Li

**Affiliations:** State Key Laboratory of Natural Medicines, School of Traditional Chinese Pharmacy, China Pharmaceutical University, Nanjing, 210009 China

**Keywords:** Cardiology, Cardiovascular diseases

## Abstract

Endothelial-to-mesenchymal transition (EndoMT), the process wherein endothelial cells lose endothelial identity and adopt mesenchymal-like phenotypes, constitutes a critical contributor to cardiac fibrosis. The phenotypic plasticity of endothelial cells can be intricately shaped by alteration of metabolic pathways, but how endothelial cells adjust cellular metabolism to drive EndoMT is incompletely understood. Here, we identified 6-phosphofructo-2-kinase/fructose-2,6-biphosphatase 3 (PFKFB3) as a critical driver of EndoMT via triggering abnormal glycolysis and compromising mitochondrial respiration. Pharmacological suppression of PFKFB3 with salvianolic acid C (SAC), a phenolic compound derived from *Salvia miltiorrhiza*, attenuates EndoMT and fibrotic response. PFKFB3-haplodeficiency recapitulates the anti-EndoMT effect of SAC while PFKFB3-overexpression augments the magnitude of EndoMT and exacerbates cardiac fibrosis. Mechanistically, PFKFB3-driven glycolysis compromises cytoplasmic nicotinamide adenine dinucleotide phosphate (reduced form, NADPH) production via hijacking glucose flux from pentose phosphate pathway. Efflux of mitochondrial NADPH through isocitrate/α-ketoglutarate shuttle replenishes cytoplasmic NADPH pool but meanwhile impairs mitochondrial respiration by hampering mitochondrial iron-sulfur cluster biosynthesis. SAC disrupts PFKFB3 stability by accelerating its degradation and thus maintains metabolic homeostasis in endothelial cells, underlying its anti-EndoMT effects. These findings for the first time identify the critical role of PFKFB3 in triggering EndoMT by driving abnormal glycolysis in endothelial cells, and also highlight the therapeutic potential for pharmacological intervention of PFKFB3 (with SAC or other PFKFB3 inhibitors) to combat EndoMT-associated fibrotic responses via metabolic regulation.

## Introduction

Cardiac fibrosis, characterized by the excessive production and deposition of scar tissue, is often a result of conditions such as hypertension and ischemic heart disease.^1^ Myofibroblasts represent primary contributors to cardiac fibrosis by prompting the deposition of excessive extracellular matrix and tissue remodeling.^2^ Although resident cardiac fibroblasts constitute the major source of activated myofibroblasts during fibrotic disorders, they can also be derived from endothelial cells (ECs) via a process termed endothelial-to-mesenchymal transition (EndoMT).^3^ Once entering EndoMT process, ECs are reprogrammed to reduce the expression of endothelial-specific proteins and meanwhile acquire mesenchymal-specific phenotypes by expressing α-smooth muscle actin (α-SMA) and secreting fibronectin and fibrillar collagens, thus initiating the fibrotic responses.^4^ Intervention of EndoMT might provide a promising avenue for therapeutic benefits,^5,6^ but up to date there is still lack of effective therapies.

Although quiescent ECs are exposed to high oxygen levels in the blood vessels, their metabolism is glycolysis-addicted, since adenosine triphosphate (ATP) is mainly generated from glycolysis in oxygen-replete conditions. Relying on glycolysis, ECs provide metabolic support for continuous vessel growth, and a recent study has observed that enhanced glycolysis drives EndoMT in renal capillary ECs during kidney fibrosis.^7^ Also, it is well accepted that glycolysis plays a crucial role in fibrotic responses since glucose metabolism can provide energy for anabolic processes and the building blocks for collagen production.^8^ This is analogous to the Warburg effects observed in cancers, wherein cancer cells preferentially utilize aerobic glycolysis to generate ATP and to provide carbon to fuel proliferation even in oxygen-rich environment.^9^

6-phosphofructo-2-kinase/fructose-2,6-biphosphatase 3 (PFKFB3) is a metabolic enzyme that generates fructose-2,6-bisphosphate (F-2,6-BP), which serves as a potent allosteric activator of the rate-controlling glycolytic enzyme phosphofructokinase-1 (PFK-1) to sustain glycolysis. Glycolysis drives angiogenesis and evidence have demonstrated the important role of PFKFB3 in manipulating endothelial phenotype transitions.^10^ PFKFB3 inhibition blocked pathological angiogenesis by reducing endothelial proliferation and migration.^11,12^ Moreover, inhibition of PFKFB3 was proven to render proliferating ECs becoming quiescent to maintain phenotypic homeostasis. Although most findings around PFKFB3 were observed in manipulation of vessel sprouting, these events also suggest the potential role of PFKFB3 in controlling EndoMT, in consideration of the phenomenon that ECs predominantly rely on glycolysis to support cell growth and differentiation.

Apart from glycolysis, ECs consume glucose for biomass synthesis partially through diversion of glycolytic intermediates to anabolic side pathways such as the pentose phosphate pathway (PPP). PPP branches from glycolysis at the first committed step of glucose metabolism to generate biomass and reducing equivalent (Nicotinamide Adenine Dinucleotide Phosphate, reduced form, NADPH) required for anabolic metabolism and redox homeostasis.^13^ Inhibition of rate-controlling enzyme of the PPP is shown to compromise EC growth and vessel sprouting.^14^ Since glycolysis and PPP pathway both competitively consumes glucose-6-phosphate (G-6-P) as metabolic substrate, the metabolic flux of PPP pathway might be influenced by different mode of glycolysis, and vice versa. Indeed, inhibition of pyruvate kinase M2, a rate-limiting enzyme of glycolysis, can divert glucose flux into PPP to enhance cellular antioxidant responses, largely due to increased NADPH production.^15^ Apart from glycolysis and PPP, mitochondrial oxidation is also involved in the progression of EndoMT,^16^ but the reciprocal links between these metabolic processes have rarely been discussed. In addition, whether and how metabolic state of ECs might affect cellular proliferation and differentiation in the context of EndoMT is an important issue to be investigated.

Salvianolic acid C (SAC) is a phenolic component derived from *Salvia miltiorrhiza* (Danshen), which has been clinically prescribed for the treatment of cardiovascular and fibrotic disorders.^17,18^ Salvianolic acids reportedly restricted endothelial permeability and inhibited vascular remodeling via suppression of inflammation and oxidative stress, contributing to ameliorated endothelial dysfunction in different disease settings.^19,20^ Recent studies have demonstrated that salvianolic acids blocked transforming growth factor-β1 (TGF-β1) and reactive oxygen species (ROS) signaling cascades to protect pulmonary arterial ECs from mesenchymal transition.^21,22^ But whether and how salvianolic acid might affect the progression of EndoMT via metabolic regulation remains elusive. Herein, we identify PFKFB3 as a crucial driver of EndoMT by triggering abnormal glycolytic/mitochondrial metabolism and disrupting intracellular NADPH distribution. SAC disrupts PFKFB3 protein stability to reroute glucose flux from glycolysis to PPP shunt, restoring NADPH abundance and maintaining activity of mitochondrial respiration. These results indicate that maintaining metabolic homeostasis in ECs might represent a feasible strategy for the designation of anti-fibrosis therapy by targeting EndoMT.

## Results

### SAC alleviates EndoMT-associated fibrotic response

**Fig. 1:**
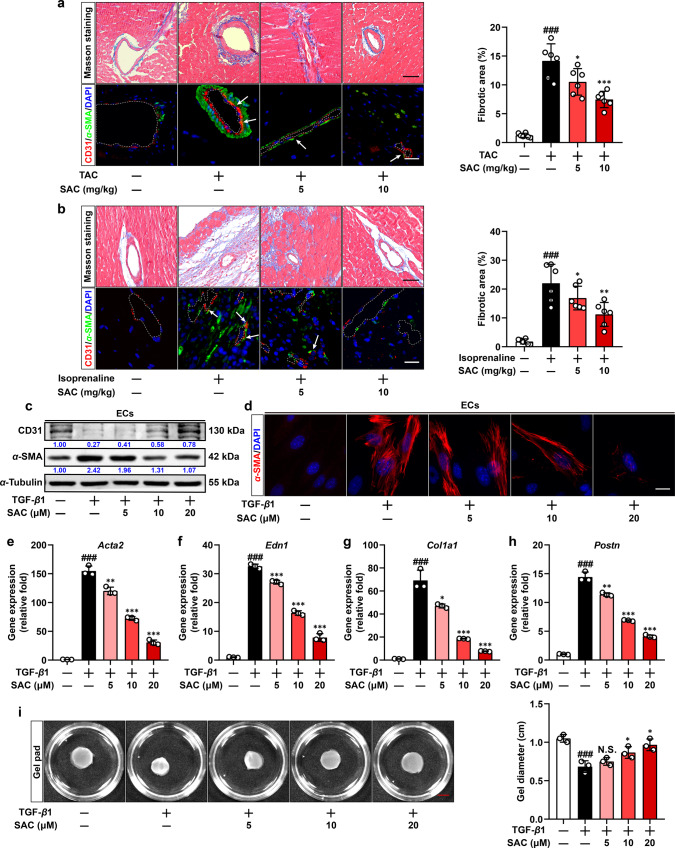
SAC alleviates EndoMT-associated fibrotic response. **a** Masson staining and immunofluorescent double-staining of CD31 (Red) and α-SMA (Green) of cardiac sections (white dotted line was applied to delineate the inner surface of blood vessel) from sham-operated, TAC-operated and SAC-treated mice and the calculated fibrotic area, scale bar, upper panel: 50 μm, lower panel: 20 μm (*n* = 6). **b** Masson staining and immunofluorescent double-staining of CD31 (Red) and α-SMA (Green) of cardiac sections from vehicle, isoprenaline and SAC-treated mice and the calculated fibrotic area, scale bar, upper panel: 50 μm, lower panel: 20 μm (*n* = 6). **c** Immunoblot analysis of CD31 and α-SMA expression in endothelial cells (ECs) treated with vehicle, TGF-β1 and SAC. α-Tubulin was used as the loading control (*n* = 3). **d** Immunofluorescent staining of α-SMA in ECs treated with vehicle, TGF-β1 and SAC, scale bar, 20 μm (*n* = 3). **e-h** q-PCR analysis of *Acta2*, *Edn1*, *Col1a1,* and *Postn* mRNA level in ECs treated with vehicle, TGF-β1 and SAC. 18s RNA was used as the internal reference (*n* = 3). **i** Representative images and the calculated gel diameter in gel pad contraction assay of ECs treated with vehicle, TGF-β1 and SAC, scale bar, 5 mm (*n* = 3). Data are represented as mean ± SD. ^###^*p* < 0.001 versus control group. *p < 0.05, **p < 0.01, ***p < 0.001, N.S., nonsignificant versus model group

Pressure-overload-induced EndoMT constitutes a pivotal contributor to cardiac fibrosis, we first examined the therapeutic effects of SAC in a pressure-overload mouse model. SAC administration (5, 10 mg/kg, i.p) effectively reduced collagen deposition in the perivascular region (Fig. 1a, upper panel). In addition, double immunofluorescent staining revealed significant induction of EndoMT, indicated by expression of interstitial marker α-SMA (Green) in CD31-positive vascular cells (Red) in mice underwent transverse aortic constriction (TAC) operation, and this pathological phenotype was attenuated after SAC treatment (Fig. 1a, lower panel). Concordantly, impairment of left ventricular functions and myocardial hypertrophy induced by pressure-overload were also reversed by SAC (Supplementary Fig. 1a–c). Therapeutic effects of SAC on perivascular fibrosis and in vivo EndoMT were also recapitulated in mice subjected to isoprenaline challenge (Fig. 1b). No significant hepatoxicity or nephrotoxicity was detected by Hematoxylin and Eosin (H&E) staining in both mouse models after SAC administration (Supplementary Fig. 2a, b). Above results collectively support that SAC protects heart against EndoMT-associated fibrotic responses.

EndoMT is a specific cellular process characterized by the loss of endothelial features and the acquisition of myofibroblast phenotype in ECs. Since TGF-β1 signaling pathway is a well-defined inducer of EndoMT, we established in vitro EndoMT model via treating ECs with TGF-β1. As a result, TGF-β1 stimulation-induced phenotype transition, indicated by reduced CD31 protein expression and upregulated protein levels of α-SMA (Fig. 1c). The formation of mature filamentous α-SMA fibers also confirmed the phenotypic transition of ECs (Fig. 1d). SAC elicited minimal effects on quiescent ECs (Supplementary Fig. 3a), but effectively reversed these alternations of CD31 and α-SMA and blunted the induction of fibrogenic genes (*Acta2*, *Edn1*, *Col1a1*, and *Postn*) in TGF-β1-treated ECs in a concentration-dependent manner (Fig. 1e–h). In addition, transdifferentiated ECs characterized by increased α-SMA expression can directly mediate fiber contraction. Thus, we used an in vitro gel contraction assay to evaluate contractile ability of ECs, and SAC blocked encapsulated ECs-mediated collagen gel pad contraction (Fig. 1i), thereby providing functional evidence to support restrained EndoMT. As a support, we isolated primary adult mouse cardiac endothelial cells (AMCECs, Supplementary Fig. 4a), and observed that SAC was also effective in ameliorating TGF-β1-induced EndoMT in AMCECs, further convincing the therapeutic efficacy of SAC (Supplementary Fig. 4b–f). We further tested whether SAC affected TGF-β1 stability, and also conducted drug affinity responsive target stabilization assay (DARTS, Supplementary Fig. 5a) to inquire whether SAC directly interacted with TGF-β1 and thus hindered its activity. Co-incubation of TGF-β1 recombinant protein with SAC did not affect the protein abundance of TGF-β1 itself (Supplementary Fig. 5a, Lane 1 and Lane 5), and SAC did not delay the degradation of TGF-β1 in the presence of pronase (Supplementary Fig. 5a, Lane 1–4). These results largely ruled out the possibility that SAC directly reduced TGF-β1 content or hindered the activity of TGF-β1 in the culture medium. Together, above results demonstrate that SAC effectively prevents transition of ECs into the mesenchymal-like phenotype.

### SAC attenuates EndoMT via restoration of imbalanced glycolytic/mitochondrial metabolism

**Fig. 2:**
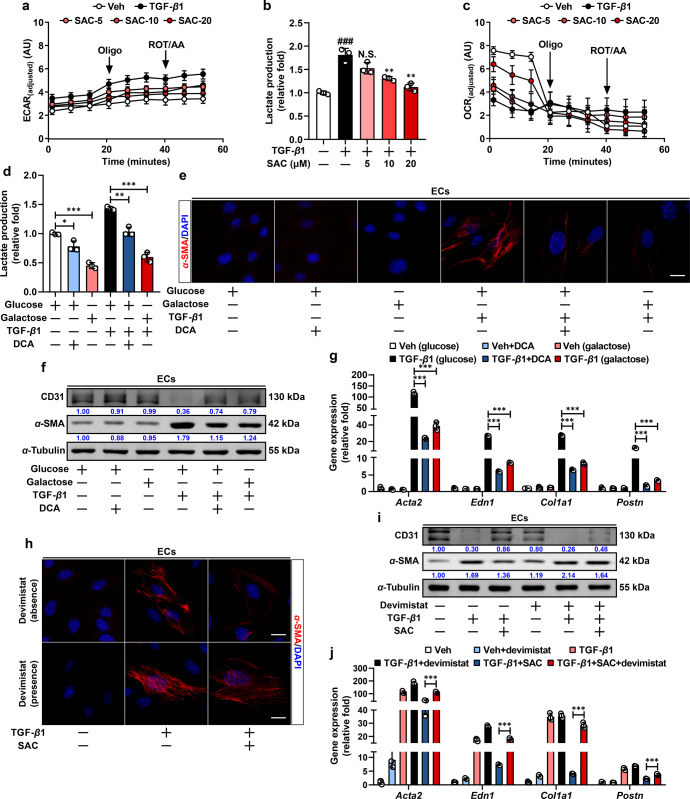
SAC attenuates EndoMT via restoration of imbalanced glycolytic/mitochondrial metabolism. **a**, **b** Analysis of ECAR and lactate production in ECs treated with vehicle, TGF-β1 and SAC (*n* = 3). **c** Analysis of OCR in ECs treated with vehicle, TGF-β1 and SAC (*n* = 3). **d** Lactate production in ECs treated with vehicle, TGF-β1, DCA, and galactose (*n* = 3). **e** Immunofluorescent staining of α-SMA in ECs treated with vehicle, TGF-β1, DCA and galactose, scale bar, 20 μm (*n* = 3). **f** Immunoblot analysis of CD31 and α-SMA expression in ECs treated with vehicle, TGF-β1, DCA and galactose. α-Tubulin was used as the loading control (*n* = 3). **g** q-PCR analysis of *Acta2*, *Edn1*, *Col1a1* and *Postn* mRNA level in ECs treated with vehicle, TGF-β1, DCA, and galactose. 18s RNA was used as the internal reference (*n* = 3). **h** Immunofluorescent staining of α-SMA in ECs treated with vehicle, TGF-β1, SAC, and devimistat, scale bar, 20 μm (*n* = 3). **i** Immunoblot analysis of CD31 and α-SMA expression in ECs treated with vehicle, TGF-β1, SAC, and devimistat. α-Tubulin was used as the loading control (*n* = 3). **j** q-PCR analysis of *Acta2*, *Edn1*, *Col1a1*, and *Postn* mRNA level in ECs treated with vehicle, TGF-β1, SAC and devimistat. 18s RNA was used as the internal reference (*n* = 3). Data are represented as mean ± SD. ^###^*p* < 0.001 *versus* control group. **p < 0.01, ***p < 0.001 versus model group (**b**) or indicated group

ECs primarily rely on glycolysis to adapt to varied circulatory conditions. TGF-β1 stimulation augmented aerobic glycolysis, indicated by increased extracellular acidification rate (ECAR) and lactate production capacity, which were normalized by SAC (Fig. 2a, b). In contrast to enhanced glycolysis, TGF-β1 stimulation impaired mitochondrial oxidative phosphorylation (OxPhos), evidenced by reduced oxygen consumption rate (OCR), which was also reversed by co-treatment with SAC in ECs (Fig. 2c). In addition, a pH-sensitive dye 2′,7′-Bis(2-carboxyethyl)-5(6)-carboxyfluorescein (BCECF) and tetramethylrhodamine, ethyl ester (TMRE) were applied to monitor the change of intracellular acidic state and mitochondrial membrane potential, and SAC expectedly prevented intracellular acidification and breakdown of mitochondrial membrane potential in response to TGF-β1 (Supplementary Fig. 6a).

To verify whether enhanced glycolysis was required for mesenchymal transition of ECs, we treated ECs with pyruvate dehydrogenase kinase (PDK) inhibitor dichloroacetate (DCA) to promote mitochondrial oxidation and switched substrate from glucose to galactose to force endothelial glucose metabolism to OxPhos instead of glycolysis.^23^ Both treatments reduced lactate generation (Fig. 2d). Despite no significant influence on quiescent cells, DCA and galactose inhibited α-SMA induction while preserved CD31 protein expression in TGF-β1-stimulated ECs (Fig. 2e, f). Consistently, DCA and galactose treatments suppressed fibrogenic genes expression and limited collagen gel contraction ability in ECs (Fig. 2g, Supplementary Fig. 7a). These results demonstrate that EndoMT is supported by enhancement in glycolytic metabolism. In contrast, pyruvate dehydrogenase (PDH) inhibition could shift cellular energy metabolism to glycolysis by blocking glucose oxidation. SAC reciprocally regulated CD31 and α-SMA protein expression and suppressed fibrotic responses in ECs, which were largely diminished by PDH inhibitor devimistat (Fig. 2h–j, Supplementary Fig. 7b). These results collectively demonstrate that intervention of glycolytic metabolism occupies an indispensable position in the anti-EndoMT effect of SAC.

### SAC restrains PFKFB3-driven glycolysis to block EndoMT

**Fig. 3:**
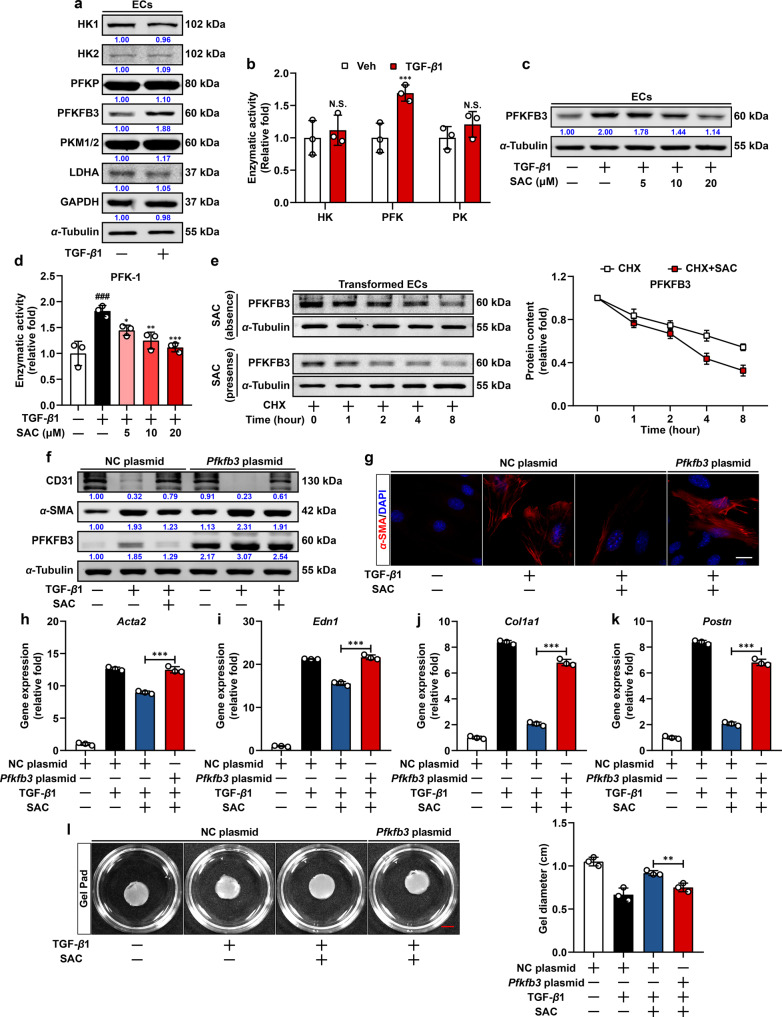
SAC blocks EndoMT via suppressing PFKFB3 expression. **a** Immunoblot analysis of HK1, HK2, PFKP, PFKFB2, PFKFB3, PKM1/2, LDHA, and GAPDH expression in quiescent and transformed ECs. α-Tubulin was used as the loading control (*n* = 3). **b** Enzymatic activities of HK, PFK-1, and PK in quiescent and transformed ECs (*n* = 3). **c** Immunoblot analysis of PFKFB3 expression in quiescent, transformed, and SAC-treated ECs. α-Tubulin was used as the loading control (*n* = 3). **d** Enzymatic activities of PFK-1 in quiescent, transformed and SAC-treated ECs (*n* = 3). **e** Immunoblot analysis of PFKFB3 expression in ECs treated with cycloheximide (CHX) in the absence or presence of SAC for different time periods. α-Tubulin was used as the loading control (*n* = 3). **f** Immunoblot analysis of CD31, α-SMA, and PFKFB3 expression in negative control (NC) or *Pfkfb3* plasmid transfected ECs following vehicle, TGF-β1 and SAC treatment. α-Tubulin was used as the loading control (*n* = 3). **g** Immunofluorescent staining of α-SMA in negative control (NC) or *Pfkfb3* plasmid transfected ECs following vehicle, TGF-β1 and SAC treatment, scale bar, 20 μm (*n* = 3). **h–k** q-PCR analysis of *Acta2*, *Edn1*, *Col1a1,* and *Postn* mRNA level in negative control (NC) or *Pfkfb3* plasmid transfected ECs following vehicle, TGF-β1 and SAC treatment. 18s RNA was used as the internal reference (*n* = 3). **l** Representative images and the calculated gel diameter in gel pad contraction assay of negative control (NC) or *Pfkfb3* plasmid transfected ECs following vehicle, TGF-β1 and SAC treatment, scale bar, 5 mm (*n* = 3). Data are represented as mean ± SD. ^###^*p* < 0.001 versus control group. *p < 0.05, **p < 0.01, ***p < 0.001 versus vehicle group (**b**), model group (**d**) or indicated group

Given the essential role of glycolysis in EndoMT, we examined the expression of three rate-limiting enzymes along with several other key molecules in glycolytic pathways. Interestingly, TGF-β1 specifically increased protein abundance of PFKFB3, while other enzymes including hexokinase 1/2, phosphofructokinase, platelet isoform, pyruvate kinase isoform M1/2, lactate dehydrogenase A, and glyceraldehyde-phosphate dehydrogenase were not significantly affected (Fig. 3a). PFKFB3 catalyzes the formation of F-2,6-BP, one of the most potent natural allosteric activators of PFK-1. Consistently, TGF-β1 stimulation increased the activity of PFK-1 to about 1.7-fold, while the activities of HK and pyruvate kinase remained relatively unchanged (Fig. 3b). To further confirm the alteration of PFKFB3 expression in ECs from different tissue, we also quantified changes in PFKFB3 protein contents in mouse bEnd.3 (brain-derived) and MS1 (islet-derived) cell lines, which reportedly underwent endothelial-to-mesenchymal transitions in response to TGF-β1 stimulation.^24–26^ As a result, we observed a consistent upregulation of PFKFB3 in both cell lines (Supplementary Fig. 8a), indicating conserved changes of endothelial PFKFB3 in response to TGF-β1 stimulation. We examined the impact of SAC on PFKFB3 and PFK-1 activity, and found that SAC attenuated PFKFB3 protein expression with a reduction in PFK-1 activity in a concentration-dependent manner (Fig. 3c, d), and these alteration of PFKFB3 and PFK-1 activity were also recapitulated in primary AMCECs (Supplementary Fig. 9a, b). Concordantly, immunofluorescent counterstaining of PFKFB3 and CD31 in fibrotic cardiac tissue from mice exposed to pressure-overload demonstrated increased PFKFB3 intensity in vascular ECs, which was also significantly reversed by SAC administration (Supplementary Fig. 9c). These results provide solid evidence for the pivotal role of PFKFB3 in EndoMT and cardiac fibrosis. In addition, when protein synthesis was inhibited by cycloheximide, SAC accelerated PFKFB3 protein degradation (Fig. 3e), suggesting that SAC impaired PFKFB3 protein stability and subsequently inactivated PFK-1. Remarkably, specific PFKFB3 kinase inhibitor PFK-015 and 3PO expectedly blunted PFK-1 activity and ECAR in ECs, and were effective in attenuating EndoMT (Supplementary Fig. 10a–f). In contrast, PFKFB3 overexpression diminished the inhibitory effects of SAC on EndoMT (Fig. 3f–l), convincing that inhibition of PFKFB3-driven glycolysis played a causal role in endothelial protection provided by SAC.

### SAC sustains PPP-derived NADPH production via inhibiting PFKFB3 expression

**Fig. 4:**
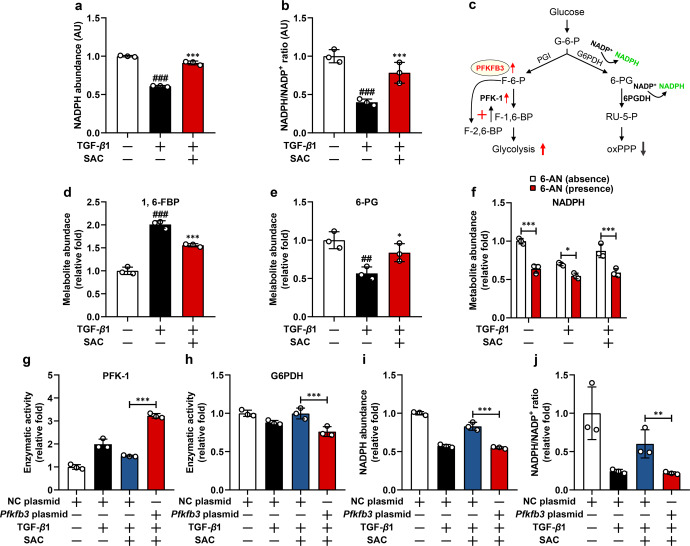
SAC increases NADPH production from pentose phosphate pathway (PPP) via downregulating PFKFB3. **a**, **b** Cellular NADPH abundance and NADPH/NADP^+^ ratio vehicle, TGF-β1 and SAC-treated ECs (*n* = 3). **c** Schematic of primary metabolic pathways for glucose flux through glycolysis and PPP. **d**, **e** Cellular F-1,6-BP and 6-PG abundance in vehicle, TGF-β1 and SAC-treated ECs (*n* = 3). **f** Cellular NADPH abundance in vehicle, TGF-β1, SAC-treated ECs in the absence or presence of 6-aminonicotinamide (6-AN) (*n* = 3). **g**, **h** Cellular PFK-1 activity, G6PDH activity, and ratio of PFK-1/G6PDH activity in negative control (NC) or *Pfkfb3* plasmid transfected ECs following vehicle, TGF-β1 and SAC treatment (*n* = 3). **i**, **j** NADPH abundance and NADPH/NADP^+^ ratio in negative control (NC) or *Pfkfb3* plasmid transfected ECs following vehicle, TGF-β1 and SAC treatment (*n* = 3). Data are represented as mean ± SD. ^##^*p* < 0.01, ^###^*p* < 0.001 versus control group. *p < 0.05, **p < 0.01, ***p < 0.001 versus model (**a**, **b**, **d**, **e**) group or indicated group

PPP, branching from glycolysis at the first committed step, consumes glucose-6-phosphate as a primary substrate and constitutes a major source of cellular NADPH. However, in contrast to enhanced glycolysis-derived lactate production, TGF-β1 reduced intracellular NADPH content and decreased the ratio of NADPH to NADP^+^, and these effects were reversed by SAC treatment (Fig. 4a, b). We speculated that PFKFB3-induced PFK-1 activation potentiated glycolytic metabolism and limited metabolic flux towards pentose shunt, which might be the reason for reduced intracellular NADPH abundance (Fig. 4c). Indeed, TGF-β1 stimulation increased fructose-1,6-Bisphosphate (F-1,6-BP) production with a corresponding reduction in 6-phosphogluconate (6-PG) generation, whereas SAC reversed these alternations, indicating a metabolic shift from PPP to glycolysis (Fig. 4d, e). In addition, the roles of SAC in the regulation of metabolites and NADPH contents were lost when PPP was blocked by glucose-6-phosphate dehydrogenase (G6PDH) inhibitor 6-aminonicotinamide (6-AN) (Fig. 4f, Supplementary Fig. 11a–c). 5-ethynyl-2′-deoxy uridine incorporation is an indicator of cell proliferation, and we found that transformed ECs exerted delayed proliferation, probably as a consequence of deficiency in PPP-derived ribose production, and 6-AN delayed cell proliferation in both quiescent and SAC-treated cells but was ineffective to transformed ECs, further implying that PPP was already impaired during EndoMT (Supplementary Fig. 11d). Interestingly, we observed that overexpression of PFKFB3 expectedly potentiated PFK-1 activity and also triggered significant reduction in G6PDH activity (Fig. 4g, h), and this phenomenon might also contribute to the impairment of PPP activity. Concordantly, PFKFB3 overexpression diminished the enhancive effect of SAC on NADPH production (Fig. 4i, j).

HK2 is a key enzyme catalyzing the first committed step of glycolysis, locating upstream pentose shunt. Notably, in contrast to PFKFB3 overexpression, HK2 overexpression in ECs increased NADPH abundance, likely due to an increase in general glucose flux (Supplementary Fig. 12a, b). Together, these results indicate that SAC preserves NADPH production by maintaining PPP activity in a PFKFB3-dependent manner.

### SAC maintains OxPhos by preserving iron-sulfur (Fe-S) cluster proteins expression

Next, we sought to investigate the molecular mechanism through which SAC maintained mitochondrial respiration in ECs. The abundance of mitochondria is controlled by a process termed mitochondrial biogenesis under strict regulation of peroxisome proliferator-activated receptor-gamma coactivator-1α (PGC-1α),^27^ and DNA content of *mt-Nd1* and *mt-Cytb* encoded by mitochondrial genomes are direct indicators of mitochondrial DNA copy numbers. Despite partial reduction in mRNA level of *Pppargc1a* (encoding PGC-1α), *mt-Nd1,* and *mt-Cytb*, TGF-β1 stimulation did not reduce mitochondrial DNA copy number and mitochondria abundance (Supplementary Fig. 13a–c), suggesting other mechanisms for SAC to preserve mitochondrial function.

**Fig. 5:**
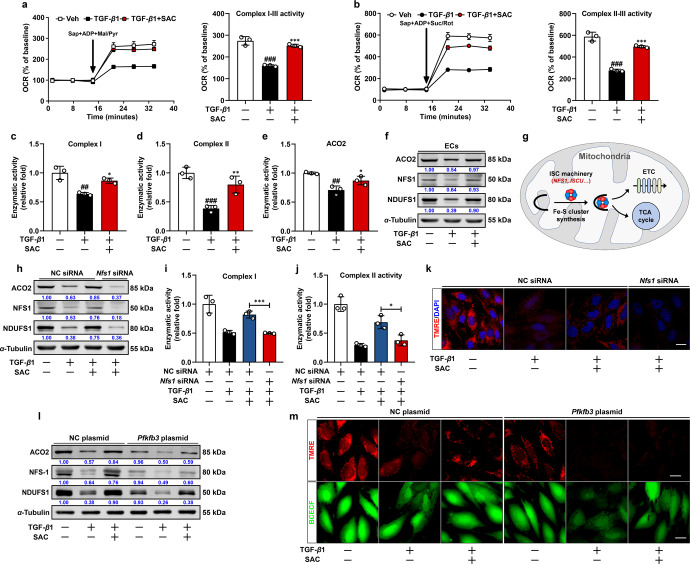
SAC maintains OxPhos by preserving Fe-S cluster protein expression. **a** Average complex I–III activity following stimulation with 10 mM pyruvate and 1 mM malate in quiescent, transformed, and SAC-treated ECs (*n* = 3). **b** Average complex II–III activity following stimulation with 10 mM succinate and 1 μM rotenone in quiescent, transformed, and SAC-treated ECs (*n* = 3). **c–e** Average complex I-NADH oxidoreductase, complex II, and ACO2 activity of quiescent, transformed and SAC-treated ECs (*n* = 3). **f** Immunoblot analysis of ACO2, NFS1 and NDUFS1 expression in quiescent, transformed, and SAC-treated ECs. α-Tubulin was used as the loading control (*n* = 3). **g** Schematic diagram of mitochondrial Fe-S cluster protein biosynthesis machinery. **h** Immunoblot analysis of ACO2, NFS1, and NDUFS1 protein expression in negative control (NC) or *Nfs1* siRNA transfected ECs following vehicle, TGF-β1 or SAC treatment. α-Tubulin was used as the loading control (*n* = 3). **i**, **j** Effect of *Nfs1* knockdown on complex I-NADH oxidoreductase, complex II activity in negative control (NC) or *Nfs1* siRNA transfected ECs following vehicle, TGF-β1 or SAC treatment (*n* = 3). **k** Effect of *Nfs1* knockdown on TMRE fluorescent staining in negative control (NC) or *Nfs1* siRNA transfected ECs following vehicle, TGF-β1 or SAC treatment, scale bar, 20 μm (*n* = 3). **l** Effect of *Pfkfb3* overexpression on ACO2, NFS1, and NDUFS1 protein expression in negative control (NC) or *Nfs1* siRNA transfected ECs following vehicle, TGF-β1 or SAC treatment. α-Tubulin was used as the loading control (*n* = 3). **m** Effect of *Pfkfb3* overexpression on TMRE and BCECF fluorescent counter-staining in negative control (NC) or *Nfs1* siRNA transfected ECs following vehicle, TGF-β1 or SAC treatment, scale bar, 20 μm (*n* = 3). Data are represented as mean ± SD. ^##^*p* < 0.01, ^###^*p* < 0.001 versus control group. *p < 0.05, **p < 0.01, ***p < 0.001 versus model group (**a**–**e**) or indicated group

Cellular Fe-S cluster proteins are the most ancient protein cofactors playing a decisive role in electron transfer devices responsible for essential and minimal function of mitochondria.^28^ Because NADPH is a critical cofactor required for the assembly of Fe-S cluster proteins,^29^ we sought to interrogated whether the reduction in NADPH abundance in ECs hampered Fe-S cluster protein function and thus compromised mitochondrial oxidative metabolism during EndoMT. Mitochondrial oxidative metabolism is dependent on a functional electron transport chain (ETC), which consists of protein complexes with resident Fe-S clusters that mediate electron transport. We first examined the activity of mitochondrial complex I and II in saponin-permeabilized ECs supplemented with respective substrates according to a previously described method.^30^ TGF-β1 dramatically reduced OCR by about 40% when exposed to complex I substrates (malate and pyruvate, Mal/Pyr), while this change was reversed by SAC (Fig. 5a). Similarly, SAC effectively restored OCR responding to complex II substrate succinate (Suc) supplemented with rotenone (Rot) (Fig. 5b). Concordantly, SAC preserved complex I/II enzymatic activities (Fig. 5c, d). Consistent with the changes in ETC complexes, activity of ACO2, a Fe-S cluster protein functioning in the tricarboxylic acid (TCA) cycle, was also reversed by SAC treatment (Fig. 5e). Immunoblot analysis revealed significant reduction in Fe-S cluster protein levels of ACO2, NDUFS1, and NFS1 in ECs post-TGF-β1 stimulation, and these alterations were restored by SAC treatment (Fig. 5f).

NFS1 is a core protein of cysteine desulfurase responsible for the biosynthesis of Fe-S cluster proteins (Fig. 5g).^31^ SAC preserved protein expression of ACO2 and NDUFS1, but the effect was blocked by *Nfs1* knockdown (Fig. 5h). As expected, SAC maintained complex I and complex II (succinate dehydrogenase, SDH) activity as well as mitochondrial membrane potential in an NFS1-dependent manner (Fig. 5i–k). These results indicate that maintenance of mitochondrial Fe-S cluster proteins represents a critical component of SAC-induced improvement of mitochondrial function during EndoMT. Notably, PFKFB3 overexpression was capable of reducing the expression of Fe-S cluster proteins and mitochondrial membrane potential and also blocked the protective effects of SAC (Fig. 5l, m). These results illustrate that SAC attenuates mitochondrial dysfunction via preserving Fe-S cluster proteins during EndoMT and also hint an implication of PFKFB3 in this process.

### SAC restores Fe-S cluster proteins via preserving cellular NADPH pool

**Fig. 6:**
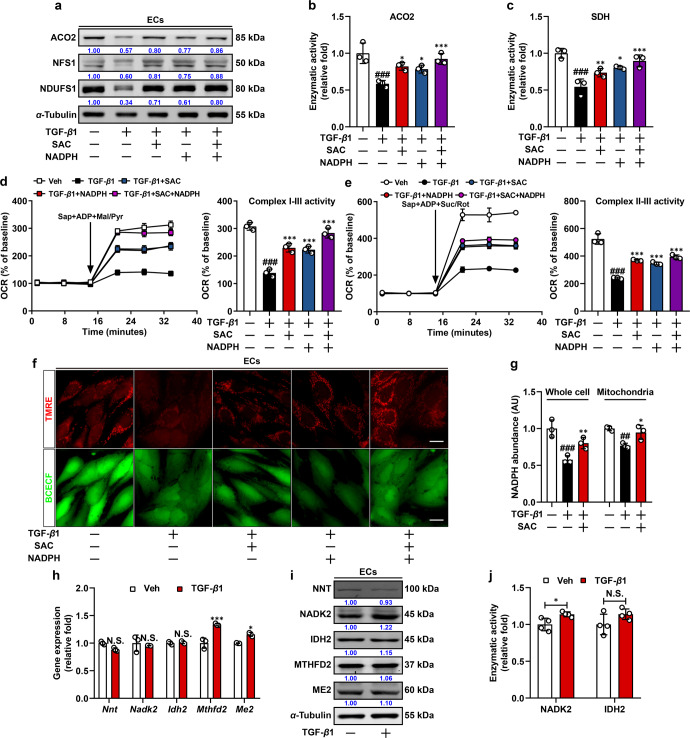
SAC restores Fe-S cluster proteins via preserving cellular NADPH pool. **a** Immunoblot analysis of ACO2, NFS1, and NDUFS1 expression in quiescent, transformed, SAC-treated, or NADPH-transfected ECs. α-Tubulin was used as the loading control (*n* = 3). **b**, **c** ACO2 and SDH enzymatic activity in quiescent, transformed, SAC-treated, or NADPH-transfected ECs (*n* = 3). **d**, **e** Average complex I–III and complex II-III activity in quiescent, transformed, SAC-treated, or NADPH-transfected ECs (*n* = 3). **f** Fluorescent counter-staining of TMRE and BCECF in quiescent, transformed, SAC-treated, or NADPH-transfected ECs (*n* = 3), scale bar, 20 μm. **g** Relative NADPH abundance in whole-cell and mitochondrial fractions in quiescent, transformed, and SAC-treated ECs (*n* = 3). **h** q-PCR analysis of *Nnt*, *Nadk2*, *Idh2*, *Mthfd2*, and *Me2* mRNA levels in quiescent and transformed ECs. 18s RNA was used as the internal reference (*n* = 3). **i** Immunoblot analysis of NNT, NADK2, IDH2, MTHFD2, and ME2 expression in quiescent and transformed ECs. α-Tubulin was used as the loading control (*n* = 3). **j** NADK2 and IDH2 enzymatic activity in quiescent and transformed ECs (*n* = 4). Data are represented as mean ± SD. ^##^*p* < 0.01, ^###^*p* < 0.001, N.S., nonsignificant versus control group. *p < 0.05, **p < 0.01, ***p < 0.001 versus model group (**b**–**e**, **g**) or control group (**h**, **j**)

Cellular NADPH homeostasis is a prerequisite for the maintenance of mitochondrial Fe-S cluster biosynthesis, we thus endeavored to test whether impaired mitochondrial Fe-S proteins resulted from deficiency in NADPH content in ECs. Since NADPH can hardly penetrate cell membrane, we performed transfection technique using an X-tremeGENE HP DNA transfection reagent to deliver NADPH into ECs (Supplementary Fig. 14a) as previously described.^32^ Similar to SAC, NADPH supplement restored Fe-S cluster proteins (Fig. 6a). ACO2 catalyzes the conversion of citrate to isocitrate and SDH mediates succinate oxidation, and both Fe-S cluster proteins cooperate to prompt substrate oxidation in TCA cycle and the ETC. SAC treatment and NADPH addition conformably increased ACO2 and SDH activities (Fig. 6b, c), contributing to increased complex I/II-derived OCR and preserved mitochondrial membrane potential (Fig. 6d–f). These results indicate that disruption of NADPH availability causally compromised mitochondrial function during EndoMT.

Cellular NADPH is physiologically coupled with glutathione pool and strictly correlated to intracellular redox state. SAC increased NADPH levels and this action also fueled GSH generation and blunted ROS production during EndoMT (Supplementary Fig. 14b,c). In consideration that Fe-S cluster activity is sensitive to changes in cellular redox status, we sought to verify whether increased intracellular ROS level also had a contribution to Fe-S cluster protein dysfunction. However, different from SAC, mitochondrial ROS scavenger mito-tempo failed to preserve abundance of Fe-S cluster proteins and improve mitochondrial function (Supplementary Fig. 14c–h), indicating that oxidative stress might not serve as an indispensable factor in mitochondrial dysregulation in the setting of EndoMT, in spite of a previous study showing the association between EndoMT and increased ROS production.^33^

It is well-established that NADPH maintains redox homeostasis and reductive biosynthesis with compartmentalized cytosolic and mitochondrial pools. Similar to NADPH distribution, the biosynthesis of Fe-S cluster proteins is also executed compartmentally and mitochondrial Fe-S biosynthesis is directly supported by mitochondrial NADPH pool. We collected whole cell lysate and also immunopurified mitochondrial fraction, followed by the examination of NADPH levels in both fractions. Both total and mitochondrial NADPH levels were decreased in transformed ECs with a slighter reduction in the mitochondrial fraction, and SAC effectively restored NADPH levels in both total cell lysate and mitochondrial fraction (Fig. 6g). We further determined mRNA and protein levels of key enzymes responsible for mitochondrial NADPH regeneration, no significant reduction of *Nnt* (encoding nicotinamide nucleotide transhydrogenase, NNT), *Nadk2* (encoding NAD kinase 2, NADK2), *Idh2* (encoding isocitrate dehydrogenase 2, IDH2), *Mthfd2* (encoding methylene tetrahydrofolate dehydrogenase 2, MTHFD2), and *Me2* (encoding malic enzyme 2, ME2) was observed in transformed ECs (Fig. 6h, i). Measurement of the enzymatic activities of NADK2 and IDH2 with commercial kits detected similar mild alterations (Fig. 6j). These results lead us to hypothesize that mitochondrial-cytoplasmic NADPH redistribution, rather than impaired in situ mitochondrial NADPH regeneration, might represent a potential reason for reduction in mitochondrial NADPH level.

### Realignment of intracellular NADPH distribution through isocitrate/α-ketoglutarate (α-KG) shuttle compromises OxPhos activity

**Fig. 7:**
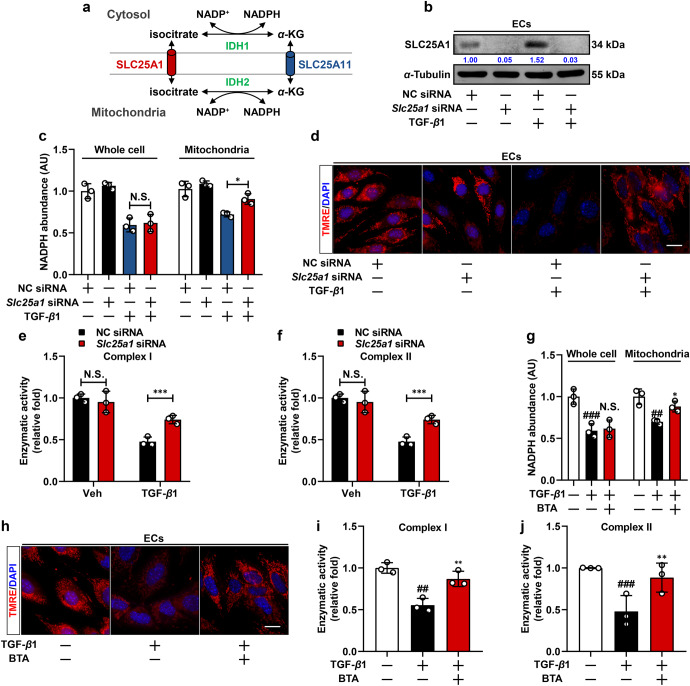
Realignment of intracellular NADPH distribution through isocitrate/α-ketoglutarate (α-KG) shuttle compromises OxPhos activity. **a** Schematic of isocitrate/α-KG shuttle machinery in connecting cytosolic and mitochondrial NADPH pool. **b** Immunoblot analysis of SLC25A1 expression in negative control (NC) or *Slc25a1* siRNA transfected ECs following vehicle, TGF-β1 or SAC treatment. α-Tubulin was used as the loading control (*n* = 3). **c** Relative NADPH abundance in whole-cell and mitochondrial fractions in NC siRNA or *Slc25a1* siRNA transfected ECs post vehicle or TGF-β1 treatment (*n* = 3). **d** Fluorescent staining of TMRE in NC siRNA or *Slc25a1* siRNA transfected ECs post vehicle or TGF-β1 treatment, scale bar, 20 μm (*n* = 3). **e**, **f** Relative complex I and complex II activity in NC siRNA or *Slc25a1* siRNA transfected ECs post vehicle or TGF-β1 treatment (*n* = 3). **g** Relative NADPH abundance in whole-cell and mitochondrial fractions in quiescent, transformed, and 1, 2, 3-benzenetricarboxylic acid hydrate (BTA)-treated ECs (*n* = 3). **h** Fluorescent staining of TMRE in quiescent, transformed, and BTA-treated ECs, scale bar, 20 μm (*n* = 3). **i**, **j** Relative complex I and complex II enzymatic activity in quiescent, transformed, and BTA-treated ECs (*n* = 3). Data are represented as mean ± SD. ^##^*p* < 0.01, ^###^*p* < 0.001 versus control group. *p < 0.05, **p < 0.01, ***p < 0.001, N.S., nonsignificant versus model group (**g**, **i**, **j**) or indicated group

As PFKFB3-driven glycolysis reduced NADPH generation from oxidative PPP, we hypothesized that reduction in mitochondrial NADPH level might be attributed to replenishment to the impaired cytoplasmic NADPH pool. Although the mitochondrial inner membrane is impermeable to NADP(H), the intercommunication between cytoplasmic and mitochondrial pools can be conducted through the isocitrate/α-KG shuttle. IDH catalyzes the two-way conversion between isocitrate and α-KG in mammalian cells. In the mitochondrial matrix, NADP^+^-dependent IDH2 converts α-KG into isocitrate by oxidizing NADPH. Then, generated isocitrate carrying the reducing equivalent is pumped into the cytosol via the citrate carrier protein (encoded by *Slc25a1* gene), where the reducing equivalent is released for NADPH regeneration through the conversion to α-KG catalyzed by IDH1, thereby facilitating NADPH redistribution to maintain a relatively balanced state (Fig. 7a). We observed increased expression of SLC25A1, the isocitrate transporter essential for isocitrate/α-KG shuttle, in response to TGF-β1 stimulation, indicating that the isocitrate/α-KG shuttle might be switched to an activated state (Fig. 7b). Thus, we knocked down *Slc25a1* (Fig. 7b) and immuno-purified mitochondria from whole cell lysate to determine NADPH abundance in both mitochondria and whole-cell fractions. As a result, *Slc25a1* knockdown effectively restored mitochondrial NADPH abundance, while total NADPH levels remained almost constant (Fig. 7c). Impaired mitochondrial membrane potential along with complex I and complex II activity were also improved by *Slc25a1* knockdown (Fig. 7d–f). We further applied a mammalian mitochondrial NADPH/NADP^+^ sensor pEBTet_NADP-Snifit-(mito) to monitor mitochondrial NADPH/NADP^+^ status wherein NADPH/NADP^+^ ratio was negatively correlated to the ratio of SiR/TMR channel intensity.^34^ In TGF-β1-stimulated ECs, fluorescent intensity ratio of SiR/TMR increased compared to quiescent ECs, indicating a decreased NADPH/NADP^+^ ratio in mitochondrial fraction, and blocking isocitrate/α-KG shuttle by *Slc25a1* knockdown was able to preserve mitochondrial NADPH/NADP^+^ state (Supplementary Fig. 15a).

Consistently, pharmacological blockage of isocitrate transport with 1, 2, 3-benzenetricarboxylic acid hydrate (BTA), the inhibitor of tricarboxylate carrier,^35^ elicited similar effects on mitochondrial NADPH abundance and oxidative metabolism (Fig. 7g–j, Supplementary Fig. 15b). These results further evidence that when cytosolic NADPH pool is impaired due to PFKFB3 induction, compensatory induction of NADPH efflux and redistribution might be triggered and responsible for reduced NADPH contents and impaired oxidative metabolism in mitochondria.

### SAC attenuates EndoMT-associated cardiac fibrosis via downregulating PFKFB3

**Fig. 8:**
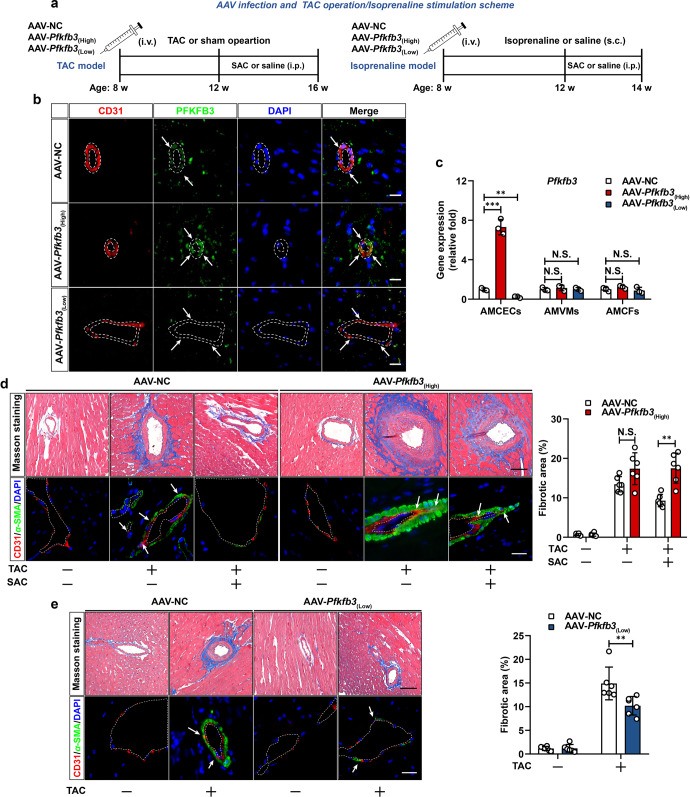
SAC attenuates EndoMT-associated cardiac fibrosis via downregulating PFKFB3. **a** Schematic for AAV-based endothelial-specific overexpression or knockdown of *Pfkfb3* and model preparation procedure in mice. **b** Immunofluorescent counter-staining of PFKFB3 (Green) and CD31 (Red) in cardiac sections (white dotted line was applied to delineate the inner surface of blood vessel) from AAV-NC, AAV-*Pfkfb3*_(High)_, and AAV-*Pfkfb3*_(Low)_ mice, scale bar, 25 μm. **c** q-PCR analysis of *Pfkfb3* mRNA level in AMCECs, AMVMs and AMCFs isolated from AAV-NC, AAV-*Pfkfb3*_(High)_, and AAV-*Pfkfb3*_(Low)_ mice. 18s RNA was used as the internal reference (*n* = 3). **d** Masson and immunofluorescent double-staining of CD31 (Red) and α-SMA (Green) of cardiac sections (white dotted line was applied to delineate the inner surface of blood vessel) from AAV-NC or AAV-*Pfkfb3*_(High)_ infected mice post sham-operation, TAC-operation, and SAC-treatment, scale bar, 50 μm (*n* = 6). **e** Masson staining and immunofluorescent double-staining of CD31 (Red) and α-SMA (Green) of cardiac sections (white dotted line was applied to delineate the inner surface of blood vessel) from AAV-NC and AAV-*Pfkfb3*_(Low)_ mice post-sham-operation, TAC-operation and SAC-treatment, scale bar, 50 μm (*n* = 6). Data are represented as mean ± SD. **p < 0.01, ***p < 0.001, N.S., nonsignificant versus indicated group

To interrogate whether inhibition of endothelial PFKFB3 underlies the therapeutic effects of SAC on EndoMT in primary AMCECs and in vivo, we generated endothelial-specific *Pfkfb3*-overexpression (AAV-*Pfkfb3*_(High)_) mice by intravenously injection of adeno-associated virus (serotype Vec, AAV-Vec, Fig. 8a). Endothelial-specific *Pfkfb3* overexpression was visualized by immunofluorescent staining of PFKFB3 protein in CD31-positive vascular cells (Fig. 8b) and also confirmed by specific upregulation of *Pfkfb3* mRNA level in isolated adult mouse cardiac endothelial cells (AMCECs) rather than in ventricular myocytes (AMVMs) or cardiac fibroblasts (AMCFs, Fig. 8c). In isolated AMCECs, SAC was effective in protecting cells isolated from AAV-NC mice against EndoMT in response to TGF-β1 exposure, but elicited minimal effects on cells from AAV-*Pfkfb3*_(High)_ mice (Supplementary Fig. 16a-d). In addition, after TAC surgery, Masson staining showed that SAC administration triggered decrease in peri-vascular collagenous fiber deposition along with reduction in colocalization of α-SMA and CD31 immunofluorescent signal in the heart, but these effects were lost in AAV-*Pfkfb3*_(High)_ mice (Fig. 8d). Concordantly, *Pfkfb3*-overexpression demonstrated similar abolishment on SAC-induced beneficial effects on mice subjected to isoprenaline challenge (Supplementary Fig. 16e), providing additional evidence to support our hypothesis.

To further inquire whether haplodeficiency of endothelial PFKFB3 suffice to recapitulate the therapeutic effects of SAC on EndoMT, we generated *Pfkfb3*-knockdown ECs by applying specific siRNA. In TGF-β1-stimulated ECs, immunoblotting showed that *Pfkfb3* knockdown expectedly attenuated PFK-1 activity (Supplementary Fig. 17a) and restored CD31 expression with corresponding decrease in α-SMA protein content (Supplementary Fig. 17b,c). Meanwhile, induction of fibrotic genes responding to TGF-β1 was also inhibited by *Pfkfb3* knockdown (Supplementary Fig. 17d–g). To further verify the critical role of PFKFB3 in driving EndoMT in primary AMCECs and in vivo, we generated endothelial-specific *Pfkfb3*-knockdown mice (AAV-*Pfkfb3*_(Low)_) by applying AAV-Vec as previously described (Fig. 8a). Concordantly, isolated AMCECs from AAV-*Pfkfb3*_(Low)_ mice exerted increased resistance to TGF-β1 stimulation compared to those isolated from AAV-NC mice (Supplementary Fig. 18a–d). In addition, in vivo results confirmed that endothelial-specific *Pfkfb3* silencing suppressed α-SMA induction and inhibited perivascular collagen deposition in heart from both TAC-operated and isoprenaline-stimulated mice (Fig. 8e, Supplementary Fig. 18e). Taken together, these results implicate abnormal endothelial PFKFB3 activation in EndoMT-associated cardiac fibrosis, and support a role for SAC (or other PFKFB3 inhibitors) to attenuate EndoMT and subsequent cardiac fibrosis by intervention of endothelial metabolism.

## Discussion

Endothelial metabolism is reprogrammed during the progression of EndoMT, and we uncover that induction of PFKFB3 drives glycolytic flux to support mesenchymal transition. It is noteworthy that rerouted intracellular NADPH distribution represents the interphase between enhanced glycolysis and impaired mitochondrial oxidation via blunting functions of mitochondrial Fe-S cluster proteins. By impairing PFKFB3 protein stability, SAC restrains glycolysis to ensure PPP-derived NADPH production, and thus preserves the functional integrity of mitochondrial Fe-S cluster proteins by maintaining mitochondrial NADPH homeostasis, contributing to amelioration of EndoMT and cardiac fibrosis.

A balanced glycolytic/oxidative metabolism is essential for ECs to maintain phenotypic and functional homeostasis, although glycolysis is considered as a major source of energy at quiescent state. In our study, greater dependence on aerobic glycolysis is observed in ECs in the setting of EndoMT. This metabolic shift might be viewed as an adaptive response to the phenotypic alternation, since rapidly growing cells like macrophages, tumor cells, etc., also adjust cellular metabolism to aerobic glycolysis to gain survival advantages.^36^ PDK inhibitor DCA has been proven to be effective in treating vascular remodeling in pulmonary hypertension, a pathological process well characterized by substantial EndoMT,^37^ but the mechanisms were previously explained as inducing apoptosis^38^ or normalizing ion transportation.^39^ In our study, we provide evidence that SAC, in accord with DCA and galactose, is effective in attenuating EndoMT process, largely due to restraining abnormal glycolysis and rebuilding OxPhos. Additionally, inversed metabolic shift by devimistat, a PDH inhibitor, abolishes the anti-EndoMT effects of SAC. On this basis, our study provides new insights that intervention of dysregulated glycolytic/oxidative metabolism might delay EndoMT progression.

PFKFB3 is a critical regulator of glycolysis by catalyzing the formation of F-2,6-BP, a potent natural allosteric activator of PFK-1. PFKFB3-driven glycolysis has been well documented in endothelial pathophysiology, including valve formation, vessel sprouting, etc.^40–42^ We demonstrate that SAC reverses PFKFB3 induction by promoting its degradation and therefore limits augmented glycolysis and EndoMT. Specific PFKFB3 kinase inhibitors PFK-015, 3PO, and endothelial *Pfkfb3* haplodeficiency conformably recapitulate the effects of SAC in suppression of EndoMT in vitro and in vivo, further supporting this rationale. These results collectively provide solid evidence illustrating that PFKFB3-driven glycolysis is critical for the development of EndoMT and EndoMT-associated cardiac fibrosis.

The ratio of glycolytic and PPP metabolite, F-1,6-BP/6-PG increases along with a reduction in total NADPH in a PFKFB3-dependent manner, and this effect is not further potentiated by 6-AN, an inhibitor of G6PDH that decides PPP activity, collectively pointing that augmentation in PFKFB3-driven glycolysis limits glucose flux towards PPP, and thus reduces PPP-derived NADPH production. This is in line with a previous finding that astrocytes with high expression level of PFKFB3 exert impaired PPP activities.^43^ However, due to the complexity of cellular metabolism, our study can not rule out the involvement of other NADPH metabolism pathways in this process, and tracing NADPH derived from specific metabolic pathways using isotopic labeling technique might help to better clarify this question in following studies. In addition, we observe that G6PDH activity is slightly downregulated in TGF-β1-stimulated ECs and more significantly inhibited by PFKFB3 overexpression, which is consistent with several previous findings that reduction of G6PDH activity correlates with activation of TGF-β1 signaling as well as increased PFKFB3 expression.^44–48^ This phenomenon might also exacerbate the impairment of PPP activity during EndoMT, despite that the underlying mechanism remains controversial. In contrast to the effects of PFKFB3, we notice that HK2 overexpression increases cellular NADPH abundance. In light of the previous finding that cardiac-specific overexpression of HK2 augments PPP flux and attenuates cardiac hypertrophy,^49^ and hexokinase activation is linked to increased PPP activity during acute kidney injury,^50^ it might be explained by the condition that HK2 lies upstream to glycolysis/PPP shunt and thus unbiasedly enhances glucose flux.

Fe-S cluster proteins are indispensable cofactors required for electron transport, and we show that the deficiency in Fe-S cluster proteins is responsible for impaired oxidative phosphorylation in ECs. These results correspond to a previous finding that compromised mitochondrial fatty acid oxidation is also engaged in EndoMT progression.^16^ The functional integrity of Fe-S cluster proteins are affected by both cellular NADPH level and redox state.^51^ NADPH replenishment restores Fe-S cluster protein content and OxPhos activity, hinting that Fe-S deficiency might be directly attributed to reduced intracellular NADPH levels. Nevertheless, although reduction in NADPH level couples increased ROS production, it is not a crucial factor involved in mitochondrial dysfunction since mito-tempo fails to rebuild Fe-S cluster protein contents and OxPhos activity.

In spite of the view that eukaryotic cells maintain compartmentalized NADPH metabolism in different organelles, the inter-communication between mitochondrial and cytoplasmic NADPH pool in different pathophysiological settings has been recently uncovered. For example, knockdown of *Me1*, an NADPH regeneration enzyme that primarily localizes in the cytoplasm is shown to trigger an increase in *Idh2* expression in mitochondrion in several tumor cell lines.^52^ In contact-inhibited fibroblasts, a flux from α-KG to citrate via IDH2 in the TCA cycle is enhanced to shuttle NADPH from the mitochondrion to cytosol for redox defense or fatty acid synthesis.^53^ In our study, we demonstrate that disrupted functions of Fe-S cluster proteins are attributed to redistribution of cytoplasmic and mitochondrial NADPH, which requires a functional IDH/α-KG shuttle. We provide experimental evidence that this distribution renders maintenance of cytoplasmic NADPH homeostasis but might also hamper NADPH-dependent reactions in mitochondria. A recent finding reveals the essential role of mitochondrial NADPH in proline biosynthesis and cell proliferation,^54^ this is consistent with our finding that impaired mitochondrial NADPH abundance is accompanied by delays in EC proliferation during EndoMT. Nevertheless, several intriguing questions remain including by what means ECs sense and adjust compartmentalized NADPH metabolism, and whether and how compensatory alteration in NADPH distribution might provide survival advantages to ECs during EndoMT.

**Fig. 9:**
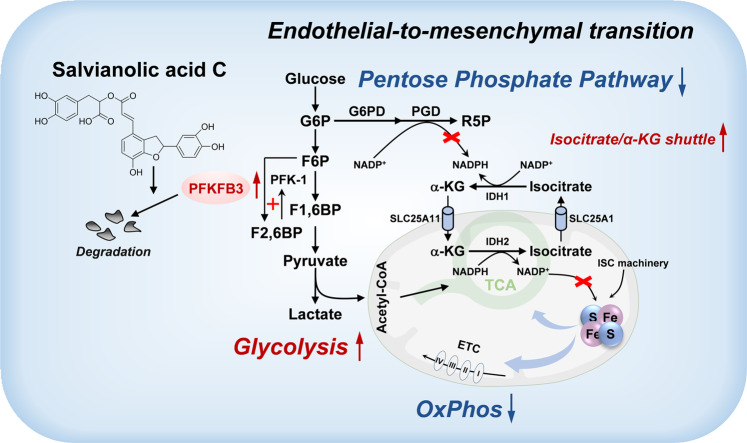
Schematic depicting the molecular mechanism through which SAC restrains EndoMT via suppression of PFKFB3. EndoMT is characterized by increased expression of PFKFB3 that drives abnormal glycolysis and hijacks glucose flux from pentose phosphate pathway (PPP) to compromise cytoplasmic NADPH production. Efflux of mitochondrial NADPH through isocitrate/α-KG shuttle replenishes cytoplasmic NADPH pool but meanwhile impairs mitochondrial respiration by hampering mitochondrial Fe-S cluster protein biosynthesis. SAC disrupts PFKFB3 stability by accelerating its degradation and thus maintains endothelial metabolic homeostasis, underlying its anti-EndoMT effects

Together, these findings provide insights into the crucial role of PFKFB3 in regulation of glucose metabolism during EndoMT. ECs intricately shift the balance between glycolysis and PPP via PFKFB3 induction and therefore impair mitochondrial metabolism via modifying compartmentalized NADPH, which underlies the metabolic basis of EndoMT (Schematic see Fig. 9). Additionally, we provide proof of concept that suppression of PFKFB3-driven glycolysis by SAC or other pharmacological agents targeting this metabolic vulnerability might represent a feasible therapeutic strategy for EndoMT-associated fibrotic disorders.

## Materials and methods

### Reagents and antibodies

Salvianolic acid C was purchased from Chengdu MUST biotechnology (Chengdu, China). Isoprenaline (I5627), 4′,6-diamidino-2-phenylindole (DAPI, D8417), heparin (H3393), Endothelial Cell Growth Supplement (ECGS, 211F-GS), glucose (G8769), verapamil (V4629), sulfapyridine (S6252), rotenone (R8875), antimycin A (A8674), digitonin (D141), malate (1613881), pyruvate (P2256), succinate (W327700), adenosine 5’-diphosphate (ADP, 1905), NADPH (NADPH-RO), Mito-tempo (SML0737), Pronase (10165921001), DNase (D-4527), HEPES (H4034), 2,3-Butanedione 2-Monoxime (BDM, 203984), Taurine (T8691) were from Sigma-Aldrich (St. Louis, MO, USA). PFK-015 (HY-12204), 3PO (HY-19824), devimistat (HY-15453), dichloroacetate (DCA, HY-Y0445A), galactose (HY-N0210), cycloheximide (HY-12320) were from MedChemExpress (Monmouth Junction, NJ, USA). 1, 2, 3-benzenetricarboxylic acid hydrate (BTA, B100864) and oligomycin (O111756) were from Aladdin (Shanghai, China). Human (5154LF) and mouse TGF-β1 (5231L) were from Cell Signaling Technology Inc. (Danvers, MA, USA). L-glutamine (25030081), antibiotics (100 unit/mL penicillin and 100 mg/mL streptomycin, 15140-122), and Laminin (Natural, Mouse, 23017-015) were from Thermo Fischer Scientific (Waltham, MA, USA). Recombinant Mouse TGF-β1 Protein (7666-MB-005) was purchased from R&D system (McKinley Place NE, MN, USA). Collagenase, type II (LS004176) and Collagenase, type IV (LS004188) were from Worthington Biochemical Cooperation (Lakewood, NJ, USA). CD31 MicroBeads, mouse (130-097-418) were from Miltenyi Biotec (Bergisch Gladbach, Germany). Puromycin Dihydrochloride (IP1280) was from Beijing Solarbio Science & Technology Co., Ltd. Hoechst 33258 (KGA211-1) was from KeyGEN BioTECH (Nanjing, China).

The information on primary antibodies applied in this study were listed in Supplementary Table 1.

### Mice

All animal procedures were performed conforming to the National Institutes of Health guidelines and approved by the Institutional Animal Care and Use Committee of China Pharmaceutical University. All mice used were male C57Bl/6j mice obtained from GemPharmatech Co., Ltd (Nanjing, China). Mice were housed and fed under standard laboratory conditions with a 12-h light/dark cycle from 6 a.m. to 6 p.m.

To generate endothelial-specific PFKFB3 knockdown or overexpression mice, recombinant AAV-Vec vectors carrying mouse *Pfkfb3* or shRNA targeting mouse *Pfkfb3* with a *Tie* promoter (AAV-Vec-*Pfkfb3*, Hanbio, Inc., Shanghai, China) were used.^55^ In brief, C57Bl/6j mice were intravenously injected with 100 μL of AAV-Vec-*Pfkfb3* virus suspension (virus titer > 10^12^ vg/mL) blend with 100 μL normal saline. Control mice were injected with an equal volume of AAV9-control suspension. Endothelial *Pfkfb3*-knockdown and overexpression was validated 4 weeks after virus injection by counterstaining PFKFB3 with CD31 of cardiac slices.

### Pressure-overload and isoprenaline animal models

Cardiac pressure overload was induced via TAC operation as previously published.^56^ Briefly, male C57Bl/6j mice (8–10 weeks, 21–24 g) were anesthetized with pentobarbital (50 mg/kg, intraperitoneally). The animals were then placed in a supine position, and the chest cavity was exposed by cutting open the proximal portion of the sternum. After the aortic arch between the innominate and left common carotid arteries was isolated, the transverse aortic arch was ligated (6-0 silk sutures) with an overlying 27-gauge needle, and then the needle was removed, leaving a discrete region of stenosis. Sham-operated mice underwent the identical surgical procedure, including isolation of the aorta, but without placement of the suture. The surgeon was blinded to the genotype/treatment. A successful aortic arch stenosis was verified by Doppler imaging of the maximum velocity of blood flow across the constriction (Vevo 3100 LT, FUJIFILM VisualSonics, Inc., Toronto, ON, Canada).

Isoprenaline-induced cardiac fibrosis model was established as previously described.^57^ Mice were subjected to subcutaneous injection of isoprenaline (50 mg/kg) once daily for 14 consecutive days, and control animals were administered with an equal volume of normal saline.

### Histological assessment of cardiac fibrosis

Heart tissues were fixed in 3.7% paraformaldehyde in Phosphate Buffer Saline (PBS), embedded in paraffin, and sectioned at 5 μm thickness. Masson’s trichrome staining was performed using standard procedures. Cardiac fibrosis was assessed in Masson’s trichrome-stained sections using Image Pro Plus (IPP) 6.0 software (Media Cybernetics, Inc., Bethesda, MD, USA). In brief, the algorithm calculates the percentage of the area covered by collagen fibers (blue) relative to the area covered by cardiac tissue (excluding airspaces). In TAC-operated mice, only fibrosis in the left ventricle was evaluated; in isoprenaline-administered mice, both ventricles were analyzed.

### Immunofluorescent staining of cardiac tissue slides

Immunofluorescent staining of cardiac tissue slides was performed as previously described.^58^ Paraformaldehyde-fixed and paraffin-embedded tissues were sectioned at 5 μm, deparaffinized in xylene, rehydrated, and blocked in PBS containing 0.1% Tween 20 and 5% goat serum (C2530-0500, VivaCell, Shanghai, China) for 1 h at room temperature. Blocked samples were incubated with mouse anti-α-SMA (1:500, ab7817, Abcam, Cambridge, UK) or Anti-PFKFB3 antibody (1:200, ab181861, Abcam) and CD31 Polyclonal antibody (1:100, 11265-1-AP, Proteintech) overnight at 4 °C followed by exposure to species-appropriate secondary antibodies anti-rabbit IgG (H&L), F(ab0)2 fragment (Alexa Fluor 488 conjugate) (1:500, 4412S, Cell Signaling Technology, Inc., Danvers, MA, USA) or goat anti-Mouse IgG (H + L) Highly Cross-Adsorbed Secondary Antibody, Alexa Fluor Plus 555 (1:500, A32727, Thermo Fischer Scientific) in 1% bovine serum album (BSA, abs9157, Absin, Shanghai, China) for 2 h at room temperature. Slides were then counterstained with nuclear dye DAPI and mounted. Images were acquired on a DeltaVision Ultra microscopic imaging system (GE Healthcare Life Science, Pittsburgh, PA, USA). Post-acquisition processing was performed equally for all representative images in a figure panel.

### Cell culture and in vitro stimulation of EndoMT

Human umbilical vein endothelial cell (HUVEC) was obtained from the American-type culture collection. Cells were cultured in 1× F-12K Medium (21127-030, GIBCO, Thermo Fischer Scientific) supplemented with 10% fetal bovine serum (FBS; 10099141, GIBCO, Thermo Fischer Scientific), heparin (0.1 mg/mL), ECGS (30 μg/mL) and antibiotics (100 unit/mL penicillin and 100 mg/mL streptomycin) at 37 °C in a humidified incubator containing 5% CO_2_ and 95% atmosphere. Mouse islet endothelial cells MS1 and mouse brain microvascular endothelial cells bEnd.3 were obtained from the National Collection of Authenticated Cell Cultures (Shanghai, China). Cells were cultured in 1× Dulbecco’s modified Eagle’s medium (DMEM; 10569044, GIBCO, Thermo Fischer Scientific) supplemented with 10% FBS and antibiotics (100 unit/mL penicillin and 100 mg/mL streptomycin) at 37 °C in a humidified incubator containing 5% CO_2_ and 95% atmosphere. In most in vitro studies, experiments were conducted on HUVEC unless otherwise noted.

In vitro stimulation of EndoMT was conducted as previously described,^59^ ECs were cultured and grown to 50% confluency and starved in media containing 3% FBS for 12 h before treatment with human TGF-β1 (10 ng/mL) for HUVEC and mouse TGF-β1 (10 ng/mL) for AMCECs, MS1, and bEnd.3 cells, for consecutive 96 h.

### Gel pad contraction assay

Gel contraction assay was performed to evaluate the contractile activity of ECs as previously published.^60^ For the gel contraction assay, 900 μL of collagen solution (1.67 mg/mL Type 1 rat collagen (354236, Corning, Bedford, MA, USA), 20 mM HEPES, 44 mM NaHCO_3_, 1× F-12K Medium) was mixed with 100 μL cells (3.3 × 10^5^ cells/mL) for a final concentration of 1.5 mg/mL collagen. Then 300 μL cell/collagen mix was plated in 48-well plate (1 × 10^5^ cells/well) and incubated for 15–20 min at 37 °C until gelled. The gel was separated from the walls of the well with the help of a 30 G needle. DMEM (600 μL /well) supplemented with 10% FBS and 10 ng/mL human TGF-β1 was added on top of the gels. Gels were scanned using a digital camera and gel diameter was measured.

### Bioenergetic analysis in intact ECs

Bioenergetic analysis in intact ECs was performed on a Seahorse XFe96 analyzer (Agilent Technologies, Santa Clara, CA, USA) using an XF Real-Time ATP Rate Assay Kit (103592-100, Agilent Technologies). Briefly, cells were seeded in the Seahorse XF96 cell culture microplates at 1 × 10^4^ cells/well/80 µL and placed back in 37 °C, 5% CO_2_ incubator. After 24 h, cells were washed in assay media made up of Seahorse XF DMEM Medium, pH = 7.4 (103576-100, Agilent Technologies) containing 10 mM glucose, 1 mM pyruvate, and 2 mM L-glutamine and incubated for 1 h in a non-CO_2_ incubator at 37 °C before a final wash in the assay media. The Seahorse XFe96 analyzer was calibrated and the assay was run using a standard XF Real-Time ATP Rate template created using the WAVE Software (V2.6.1, Agilent Technologies) and assay standard drug injections were used of 1.5 µM oligomycin in port A and 0.5 µM rotenone/antimycin A in port B.

### Measurement of intracellular NADPH abundance

The extraction protocol for NADPH and NADP^+^ was based on a previously described research.^61^ ECs were cultured in 100 mm petri dishes. After indicated pretreatment, ~1 × 10^7^ cells were washed in ice-cold PBS and immediately quenched in 1 mL of 80% methanol–20% deionized water. Cells were scraped on dry ice and cleared by centrifugation at 4 °C. Cleared supernatant was transferred to Eppendorf tube, dried under vacuum using a CentriVap concentrator (Labconco, Kansas City, MO, USA), resuspended in NADPH extraction buffer, and NADPH and NADP^+^ abundance was determined by a commercial NADP/NADPH Assay Kit (ab65349, Abcam) following the manufacturers’ instructions.

### siRNA and plasmid transfection

For RNAi experiments, siRNA targeting *Pfkfb3*, *Nfs-1*, and scrambled siRNA (GenePharma, Shanghai, China, siRNA sequences were listed in Supplementary Table 2) were used. For a single well of a six-well plate, 100 pmol siRNA and 10 μL Xfect RNA transfection polymer (631450, Takara, Kyoto, Japan) were added to 200 μL Xfect reaction buffer, mixed thoroughly, and placed at room temperature for 10 min to generate a transfection mixture. For transfection, culture medium was replaced with antibiotic-free DMEM and transfection mixture was added. The plate was incubated at 37 °C for 4 h and transfection complexes were removed and replaced with fresh media. Analysis of knockdown efficiency and other experiments were conducted 24 h post-transfection.

For plasmid transfection, plasmid overexpressing *Pfkfb3* (NCBI gene ID: 5209, pcDNA 3.1) from Public Protein/Plasmid Library (PPL, Nanjing, China) was used. For a single well of a six-well plate, 2.5 μg plasmid and 0.75 μL Xfect RNA Transfection Polymer (631317, Takara) were added to 100 μL Xfect reaction buffer, mixed thoroughly, and placed at room temperature for 10 min to generate a transfection mixture. For transfection, culture medium was replaced with antibiotic-free DMEM and transfection mixture was added. The plate was incubated at 37 °C for 4 h and transfection complexes were removed and replaced with fresh media.

### Measurement of mitochondrial DNA (mtDNA) copy number

mtDNA copy number assays were performed as previously described.^8^ DNA was collected from ECs using the SPARKeasy Blood/Tissue/Cell DNA Kit (Shandong Sparkjade Biotechnology Co., Ltd.). Analysis of mitochondrial copy number in ECs was carried out by real-time PCR method using mitochondrion-encoded reduced nicotinamide-adenine dinucleotide dehydrogenase 1 (*mt-Nd1*) and mitochondrial cytochrome b (*mt-Cytb*) as the mtDNA markers and *Ndufs1* as a genomic marker. The primer pairs used were listed in Supplementary Table 3.

### RNA extraction and q-PCR experiments

Total RNA from cell samples was isolated using FineProtect Universal RNA Kit (R203, Genfine Biotech Co., Ltd., Changzhou, China) and reversed transcribed using a Evo M-MLV Mix Kit with gDNA Clean for qPCR (AG11728, Accurate Biotechnology Co., Ltd, Hunan, China) according to the manufacturers’ instructions. q-PCR experiment was performed in a 20 μL reaction system, which contains 10 μL AceQ qPCR SYBR green master mix (Q121-1, Vazyme Biotech), 7 μL H_2_O, 2 μL cDNA, and 1 μL primer, using the LightCycler480 q-PCR system (Roche Molecular Systems, Inc., Basel, Switzerland). 18s RNA was used as internal reference and relative gene expression was expressed as the relative fold of expression level in control group. The primer sequences for *Acta2*, *Col1a1*, *Edn1*, *Postn*, and *18s* (GENEWIZ, Suzhou, China) were listed in Supplementary Table 3.

### Western blot analysis

Protein concentration was measured with BCA Protein Assay Kit (C503021, Sangon Biotech, Shanghai, China). 20 μg protein per sample was resolved on polyacrylamide gels and transferred to 0.22 μm nitrocellulose filter membranes. Membranes were blocked with 5% nonfat milk (E-BC-R337, Elabscience Biotechnology Co., Ltd.) in Tris-buffered saline (TBS) for 1 h at room temperature, and then with primary antibody in TBS supplemented with 3% BSA overnight at 4 °C. Membranes were washed with TBST (3 × 10 min), then incubated with the IRDye® 800CW Goat anti-Rabbit IgG Secondary Antibody or IRDye® 680RD Goat anti-Mouse IgG Secondary Antibody (926-32211 and 926-68070, LI-COR Biosciences, Lincoln, NE, USA) in TBS at room temperature for 2 h and washed again. Protein imaging was performed on an odyssey Sa Imaging System (LI-COR Biosciences) and quantification was conducted on an Image Studio Lite software (Ver 5.2, LI-COR Biosciences).

### Statistical analysis

Statistical analyses and graphing were performed using GraphPad Prism (v.8.0, GraphPad Software, La Jolla, CA, USA). To assess statistical significance, comparisons between two groups were performed using an unpaired, two-tailed Student’s *t* test, and multiple-group comparisons were performed using one-way or two-way analysis of variances with Bonferroni correction. Results are presented as the mean ± SD and *p* values < 0.05 were considered to be statistically significant.

## Supplementary information

Supplementary manuscript (SIGTRANS-05963R1)

## Data Availability

All data supporting the findings of this study are available from the corresponding author on reasonable request.
