# Supplementary material for: Suppression of PFKFB3-driven glycolysis restrains endothelial-to-mesenchymal transition and fibrotic response
Source: Signal Transduct Target Ther. 2022 Sep 1;7:303. doi: 10.1038/s41392-022-01097-6 (PMC9433407; doi:10.1038/s41392-022-01097-6)
Supplement: Supplementary file 1 — Supplementary manuscript (SIGTRANS-05963R1) [file 41392_2022_1097_MOESM1_ESM.docx]

Supplementary Materials for

Suppression of PFKFB3-driven glycolysis restrains endothelial-to-mesenchymal transition and fibrotic response

Hao Zeng^1^, Ting Pan^1^, Meiling Zhan, Renaguli Hailiwu, Baolin Liu, Hua Yang*, Ping Li*

Correspondence to: [liping@cpu.edu.cn](mailto:liping@cpu.edu.cn); yanghuacpu@126.com

**This PDF file includes:**

Materials and Methods

Figures. S1 to S18

Tables S1 to S3

**Materials and Methods**

**Isolation and identification of primary adult mouse cardiac endothelial cells (AMCECs), ventricular myocytes (AMVMs) and cardiac fibroblasts (AMCFs)**

Isolation of AMCECs, AMVMs and AMCFs were performed as previously described with minor modification[^1-3^](#_ENREF_1). C57Bl/6j mice were anaesthetized and the chest was opened to excise the heart, followed by acute injection of EDTA buffer (130 mM NaCl, 5 mM KCl, 0.5 mM NaH_2_PO_4_, 10 mM HEPES, 10 mM glucose, 10 mM BDM, 10 mM taurine, 5 mM EDTA in ultrapure (18.2 MΩ.cm) H_2_O, pH=7.8) into the right ventricle to inhibit cardiomyocytes contraction. Then, heart was immediately loaded on a Langendorff perfusion system (Radnoti, Covina, CA, USA), and digestion was achieved by continuous perfusion of the heart with perfusion buffer (130 mM NaCl, 5 mM KCl, 0.5 mM NaH_2_PO_4_, 10 mM HEPES, 10 mM glucose, 10 mM BDM, 10 mM taurine in ultrapure (18.2 MΩ.cm) H_2_O, pH=7.8, temperature was maintained at 37°C using a built-in water bath system) containing 0.5 mg/mL Collagenase, type II (LS004176, Worthington), 0.5 mg/mL Collagenase, type IV (LS004188, Worthington) and 7.5 μg/mL DNase (D-4527, Sigma Aldrich) for 40-50 minutes. After digestion, chambers (atria, LV, RV) were separated and gently pulled into 1 mm pieces using forceps. Cellular dissociation was completed by gentle trituration and enzyme activity was inhibited by addition of 5 mL stop buffer (made with perfusion buffer containing 5% sterile FBS). Cell suspension was passed through a 100 μm filter, and cells underwent 4 sequential rounds of gravity settling and 3 rounds of calcium re-introduction to restore calcium concentration to physiological levels. The cell pellet in each round was enriched for AMVMs and ultimately formed a highly pure AMVMs fraction, while the supernatant from each round was combined to produce a fraction containing non-myocyte cardiac populations.

For subsequent isolation of AMCECs and AMCFs, the non-myocyte fraction was incubated with mouse CD31 microbeads (130-097-418, Miltenyi Biotec) at 4°C for 15 minutes, AMCECs were enriched by loading of microbeads-cells complex on a MACS column and placed on a magnetic stand, wherein AMCFs were firstly eluted with an elution buffer (0.5% bovine serum albumin (BSA) and 2 mM EDTA in phosphate-buffered saline (PBS), pH=7.2) while AMCECs were retained in MACS column on the magnetic stand. A secondary elution procedure was applied by pushing a plunger to flush out the magnetically labeled cells to acquire AMCECs.

AMCECs, AMVMs and AMCFs were identified by immunofluorescent staining of CD31 (endothelial marker), α-Actinin (cardiomyocyte marker) and Vimentin (fibroblast marker) in different cell populations, respectively.

**Echocardiography**

Echocardiography was performed at 4 weeks after TAC operation with the Vevo 3100 LT imaging system (Visual Sonics Inc., Toronto, Ontario, Canada) and a 30-MHz probe was applied to measure left ventricular (LV) end-diastolic diameter (LVEDD), LVend-systolic diameters (LVESD), anterior wall diastolic thickness (AWDT), anterior wall systolic thickness (AWST), posterior wall diastolic thickness (LVPWDT), and posterior wall systolic thickness (PWST) at M-Mode. Fractional shortening (FS), ejection fraction (EF), and left ventricular mass (LV mass) were calculated using Visualsonics workstation suite (version 5.6.0; VevoLab).

**Drug affinity-responsive target stabilization assay (DARTS)**

The DARTS assay was conducted as previously described with minor modifications[^4^](#_ENREF_4). In brief, approximately 2 μg of recombinant TGF-β1 protein were added into each 1.5-mL eppendorff tube, and indicated concentrations of salvianolic acid C (SAC, diluted in 1× TNC buffer, 50 mM Tris, 50 mM NaCl, 10 mM CaCl_2_, pH=7.4) was supplemented into the recombinant TGF-β1 protein. Then, to allow sufficient ligand-protein target binding, samples were gently mixed followed by incubation for 1 hour at room temperature. Recombinant TGF-β1 protein was then digested by pronase at a 1:1000 ratio (wt/wt) for precisely 30 minutes. Then, loading buffer was added into the samples and the samples were incubated at 95°C for 10 minutes. The acquired samples were then subjected to Western blot assay for determination of TGF-β1 protein abundance.

**Protein stability assay**

PFKFB3 protein stability was measured using cycloheximide (CHX) pulse chase assay as previously described[^5^](#_ENREF_5). Endothelial cells (ECs) were treated with protein synthesis inhibitor CHX (25 μM) in the absence or presence of salvianolic acid C (SAC). PFKFB3 protein levels were determined in different time points (1, 2, 4, and 8 hours) and expressed as relative fold to time 0. α-Tubulin (1:2000) was used as loading control.

**Immunofluorescent staining of α-SMA in ECs**

Cells were fixed with 3.7% paraformaldehyde diluted in warm PBS for 15 minutes, followed by permeabilizing and blocking in blocking buffer (5% normal goat serum and 0.3% Triton X-100 in PBS) for 60 minutes. Immunostaining was performed by incubation with specific α-SMA antibody (1:500) at 4°C overnight. Goat anti-Mouse IgG (H+L) Highly Cross-Adsorbed Secondary Antibody, Alexa Fluor Plus 555 (1:500, A32727, Thermo Fischer Scientific) was used as secondary antibody. Cells were counterstained with DAPI (1:1000) and mounted with Vectashield antifade mounting medium (H-1000, Vector Laboratories, Burlingame, CA, USA) for fluorescent microscopic analysis using a DeltaVision Ultra microscopic imaging system (GE Healthcare Life Science).

**Measurement of extracellular lactate production**

Extracellular lactate production was measured enzymatically in 96-well plates according to manufacturer’s specifications of Lactate-Glo Assay (J5022, Promega, Madison, WI, USA). Briefly, 1 × 10^4^ cells/well were seeded in a 96-well plate. After compound incubation, medium was collected and incubated with 30 μL detection reagent, and extracellular lactate abundance was measured with a luminometer followed by background subtraction from fresh medium. Lactate levels were normalized to protein content measured using a BCA Protein Assay Kit (23225, Thermo Fischer Scientific) from duplicate plates after lysing cells with RIPA lysis buffer (G2002, Servicebio, Wuhan, China).

**Fluorescent staining of** **tetramethylrhodamine, ethyl ester (TMRE), BCECF, CellROX and Mitotracker**

ECs cultured in glass-bottom dishes were washed with Hank's Balanced Salt Solution (HBSS, H1025, Solarbio, Beijing, China) and stained by corresponding dyes. Briefly, determination of mitochondrial membrane potential and intracellular acidic status was performed by counter-staining of TMRE (T669, Thermo Fischer Scientific) and pH indicator BCECF-AM (B1170, Thermo Fischer Scientific). The cells were labeled with TMRE and BCECF-AM dye for 30 minutes. Intracellular ROS level was monitored using a fluorescent reactive oxygen species (ROS) indicator CellROX Green Reagent (C10444, Thermo Fisher Scientific). And for measurement of mitochondrial density, cells were loaded with MitoTracker® Green FM (M7514, Thermo Fisher Scientific) following the manufacturer’s protocols. After staining, the cells were washed with HBSS for several times. Fluorescent images were captured with a DeltaVision Ultra microscopic imaging system (GE Healthcare Life Science).

**Measurement of hexokinase (HK), phosphofructokinase (PFK) and Pyruvate Kinase (PK) enzymatic activities**

Enzymatic activities of HK, PFK and PK were measured with commercial Hexokinase Activity Assay Kit (ab136957, Abcam), 6-Phosphofructokinase Activity Assay (ab155898, Abcam) and Pyruvate Kinase Assay kit (ab83482, Abcam) following the manufacturers’ instructions, respectively. Briefly, ECs were seeded in 100 mm petri dishes. After indicated treatment, cell pellets were collected and total cell protein was extracted by homogenization in assay buffer. After centrifugation to remove any insoluble debris, supernatant was transferred to a pre-chilled eppendorff and 25 μL per sample was loaded in each well of a 96-well plate. For measurement of HK activity, 50 μL of respective reaction mix was added to each well and incubated at room temperature for 40 minutes. Absorbance was measured at 450 nm. For measurement of PFK activity, 50 μL of respective reaction mix was added to each well and incubated at 37°C for 40 minutes. Absorbance was measured at 450 nm. For measurement of PK activity, 50 μL of respective reaction mix was added to each well and incubated at 25°C for 30 minutes. Absorbance was measured at 570 nm. Both HK, PFK, PK activities were calculated and expressed as relative fold to control group.

**Measurement of glucose 6 phosphate dehydrogenase (G6PDH) activity**

Enzymatic activities of G6PDH were measured with commercial Glucose 6 Phosphate Dehydrogenase Assay kit (ab102529, Abcam) following the manufacturers’ instructions. Briefly, ECs were seeded in 100 mm petri dishes. After indicated treatment, cell pellets were collected and total cell protein was extracted by homogenization in assay buffer. After centrifugation to remove any insoluble debris, supernatant was transferred to a pre-chilled eppendorff and 25 μL per sample was loaded in each well of a 96-well plate. 50 μL of respective reaction mix was added to each well and incubated at 37°C for 30 minutes. Absorbance was measured at 450 nm and G6PDH activity was calculated and expressed as relative fold of control group.

**Measurement of mitochondrial complex I and complex II activities**

Measurement of mitochondrial complex I and complex II activities was performed by two different techniques, either by measuring oxygen consumption rate (OCR) in permeabilized ECs or by measuring the enzymatic activities with commercial enzymatic assay kits.

Permeabilized cell experiments were conducted as previously described[^6^](#_ENREF_6). Culture medium was transferred to a mitochondrial assay buffer (70 mM sucrose, 220 mM mannitol, 10 mM KH_2_PO_4_, 5 mM MgCl_2_, 2 mM HEPES, 1 mM EGTA, and 0.2% [wt/vol] fatty acid-free bovine serum albumin, pH=7.2) before OCR measurements. Digitonin (30 μg/mL) was co-injected with 1 mM ADP and the indicated mitochondrial substrates to initiate permeabilization and ADP-stimulated respiration in mitochondrial assay buffer. Substrate combinations for complex I-linked respiration consisted of malate (Mal, 5 mM) plus pyruvate (Pyr, 5 mM). A combination of succinate (Suc, 5 mM) plus rotenone (Rot, 1 μM) was used to assay complex II-dependent respiration. Oxygen consumption rate measurement was performed using a XFe96 analyzer (Agilent Technologies). Mitochondrial complex I and complex II activities were calculated and expressed as % of baseline OCR after substrate addition.

Measurement of complexes I and complex II enzymatic activities was conducted using Complex I and Complex II Enzyme Activity Microplate Assay kits (ab109721 and ab109908, Abcam) according to the manufacturer’s instructions, respectively. ECs were seeded in 100 mm petri dishes. After indicated treatment, total cell protein was extracted using a detergent and the concentration was adjusted to 5 μg/μL. For measurement of complex I activity, 500 μg of cell lysate was loaded in each well of the provided plate and incubated at room temperature for 3 hours for immunocapturing. After washings, 200 μL of assay solution was added to each well and the oxidation of NADH (provided by the kit) to NAD^+^ was coupled with the simultaneous reduction of a provided dye. Absorbance was measured at 450 nm and complex I activity was calculated and expressed as relative fold to control group. For measurement of complex II activity, 250 μg of cell lysate was loaded in each well of the provided plate and incubated at room temperature for 2 hours for immunocapturing. After washings, 200 μL of activity solution was added to each well and complex II-supported production of ubiquinol was coupled with the reduction of the provided dye. Complex II activity was measured as a decrease in absorbance at 600 nm and expressed as relative fold to control group.

**Nicotinamide Adenine Dinucleotide Phosphate (reduced form, NADPH) transfection**

NADPH transfection was performed as previously described[^7^](#_ENREF_7). ECs were seeded into a six-well or 60-mm dish. Before transfection, 5 μM NADPH was diluted with 200 μL Opti-MEM (31985070, Thermo Fisher Scientific) in the sterile tube and mixed gently. Afterwards, 5 μL X-tremeGENE HP DNA Transfection Reagent (6366236001, Roche Molecular Systems) was pipetted into the tube without directly contacting with plastic walls. The transfection complex was subsequently gently mixed and incubated for 15-30 minutes at room temperature and then added into cells in a drop-wise manner. Cellular NADPH levels were measured after 24-48 hours of incubation.

**Measurement of** **fructose-1,6-bisphosphate (F-1,6-BP) and 6-phosphogluconate (6-PG) abundance**

Measurement of F-1,6-BP and 6-PG was conducted using Fructose-1,6-Bisphosphate Assay Kit (ab284537, Abcam), 6 Phosphogluconate Assay Kit (ab211071, Abcam) according to the manufacturers’ instructions, respectively. Briefly, cell pellets were homogenized in assay buffer. After centrifugation to remove any insoluble debris, supernatant was transferred to a pre-chilled Eppendorf tube and 25 μL per sample was loaded in each well of a 96-well plate. For measurement of F-1,6-BP, 50 μL of reaction mix was added to each well and incubate at 37°C for 40 minutes. Fluorescent intensity was measured at Ex/Em = 535/587 nm in end point mode. For measurement of 6-PG, 50 μL of reaction mix was added to each well and incubated at 37°C for 1 hour. Absorbance was measured at 450 nm. Both F-1,6-BP and 6-PG abundance was calculated and expressed as relative fold to control group.

**Measurement of mitochondrial aconitase 2 (ACO2) activities**

ECs were seeded in 100 mm petri dishes. After indicated treatment, mitochondrial fractions were extracted using a Mitochondria Isolation Kit for Cultured Cells (ab110170, Abcam) from cells pellets resuspended in buffer A. ACO2 activity was measured using the Aconitase Enzyme Activity Microplate assay kit (ab109712, Abcam). Protein concentration of samples were diluted to 0.3 mg/mL, and 55 μL of each were added to 5 μL aconitase solution in a clear-bottom 96-well plate. Absorbance was measured at 240 nm and enzymatic activity was calculated and expressed as relative fold to control group.

**Measurement of GSH/GSSG content**

Determination of GSH and GSSG was carried out using GSSG/GSH Quantification Kit II (G263, Dojindo Laboratories, Kumamoto, Japan) according to the manufacturer's protocol. Briefly, ECs were cultured in 100 mm petri dishes. After indicated pretreatment, cells were harvested, resuspended in 5% sulfosalicylic acid, and vortexed (4 × 1 minute) with glass beads, incubated on ice for 15 minutes, and then centrifuged for 10 minutes at 17900 × g. The supernatant was then subjected to glutathione measurement assay.

**Immuno-purification of mitochondria**

Rapid immuno-purification of mitochondria was performed following the published methodology[^9^](#_ENREF_9). In brief, ECs were engineered to express the HA-tagged OMP25 protein (83356, Addgene, Cambridge, MA, USA). After cells were exposed to indicated treatment, 1 × 10^6^ cells were washed and Dounce homogenized in KPBS (136 mM KCl and 10 mM KH_2_PO_4_, pH=7.2). The homogenate was then cleared by centrifugation and the supernatant was applied to anti-HA beads (88837, Thermo Fisher Scientific) and incubated with rotation for 3.5 minutes. The resultant beads were washed with KPBS, and methanol: water (80:20) was used to elute mitochondria for NADP(H) measurements.

**Measurements of mitochondrial NAD kinase (NADK2) and isocitrate dehydrogenase (IDH2) activities**

Enzymatic activities of NADK2 and IDH2 of ECs were measured with commercial kits following the manufacturers’ instructions. Briefly, ECs were seeded in 100 mm petri dishes. After indicated treatment, cells were collected and mitochondrial fractions were immunopurified as previously described. The mitochondria fraction was collected and used for protein quantification and measurement of enzyme activities. NADK2 and IDH2 activity were measured with NAD Kinase assay kit (A117-1-1, Jiancheng Bioengineering Institute, Nanjing, China), Isocitrate Dehydrogenase Activity Assay Kit (Sigma, MAK062) respectively, and enzyme activity was calculated and expressed as relative fold to control group.

***In vitro* monitoring of mitochondrial NADPH/NADP^+^ level with semisynthetic biosensors**

Tracing mitochondrial NADPH/NADP^+^ ratio was conducted as previously described[^10^](#_ENREF_10). ECs were transfected with the pEBTet expression vectors (113919, Addgene) using Lipofectamine 3000 (L3000015, Thermo Fisher Scientific) according to the manufacturer’s instruction. 48 hours after transfection, the cells were selected with the full growth medium supplemented with 1 μg/mL puromycin for one week. After selection, the amplified transfected cells were continuously maintained in selective conditions. Expression of the sensor proteins were induced with 10 ng/mL doxycycline for 24 hours, after which the cells were labelled with fluorescent substrates CP-TMR-SMX and SiR-Halo (1 μM) in fresh pre-warmed full growth medium supplemented with verapamil (10 μM) overnight at 37°C, 5% CO_2_. Then, cells were washed for three times with full growth medium followed by 2-hour incubation at 37 °C, 5% CO_2_. The cells were imaged before and after being treated with sulfapyridine (2 mM) to fully open the sensors, on a DeltaVision Ultra imaging system (GE Healthcare) at 37 °C, 5% CO_2_.

**EdU incorporation assay**

The EdU incorporation assay was performed according to the manufacturer’s instructions (BeyoClick™ EdU Cell Proliferation Kit with Alexa Fluor 488, C0071S, Beyotime Biotechnology, Shanghai, China). Briefly, cells were cultured in coverglass-bottom dish and 10 μM EdU was added to each dish. After additional 4 hours culture, cells were subsequently fixed with 3.7% paraformaldehyde, permeabilized in 0.3% Triton X-100/PBS, incubated in freshly made Click-iT reaction cocktail, and then stained with Hoechst 33258 (KGA211-1, KeyGEN BioTECH, Nanjing, China) for nuclear labeling. At last, samples were stored in PBS and images were acquired using a DeltaVision Ultra microscopic imaging system (GE Healthcare Life Science). The EdU incorporation rate was expressed as the percentage of EdU-positive cells to total Hoechst-positive cells. Experiments were performed in triplicate.

**References**

1. M. Ackers-Johnson, et al. A simplified, Langendorff-free method for concomitant isolation of viable cardiac myocytes and nonmyocytes from the adult mouse heart. *Circ. Res.* **119**, 909-920 (2016).

2. D. Gunduz, C.W. Hamm, M. Aslam. Simultaneous isolation of high quality cardiomyocytes, endothelial cells, and fibroblasts from an adult rat heart. *J. Vis. Exp.* (2017).

3. J.R. van Beijnum, et al. Isolation of endothelial cells from fresh tissues. *Nat. Protoc.* **3**, 1085-1091 (2008).

4. X.X. Wu, et al. Protocatechuic aldehyde protects cardiomycoytes against ischemic injury via regulation of nuclear pyruvate kinase M2. *Acta Pharm. Sin. B* **11**, 3553-3566 (2021).

5. T.H. Lee, et al. Fibroblast-enriched endoplasmic reticulum protein TXNDC5 promotes pulmonary fibrosis by augmenting TGF beta signaling through TGFBR1 stabilization. *Nat. Commun.* **11**, 20 (2020).

6. L.F. Jiang, et al. HIF-1 alpha preconditioning potentiates antioxidant activity in ischemic injury: the role of sequential administration of dihydrotanshinone I and protocatechuic aldehyde in cardioprotection. *Antioxid. Redox Signal.* **31**, 227-242 (2019).

7. W. Li, et al. NADPH levels affect cellular epigenetic state by inhibiting HDAC3-Ncor complex. *Nat. Metab.* **3**, 75-89 (2021).

8. Y. Saito, et al. DNase II activated by the mitochondrial apoptotic pathway regulates RIP1-dependent non-apoptotic hepatocyte death via the TLR9/IFN-beta signaling pathway. *Cell Death Differ.* **26**, 470-486 (2019).

9. J. Zhu, et al. Mitochondrial NADP(H) generation is essential for proline biosynthesis. *Science* **372**, 968-972 (2021).

10. O. Sallin, et al. Semisynthetic biosensors for mapping cellular concentrations of nicotinamide adenine dinucleotides. *Elife* **7**, (2018).


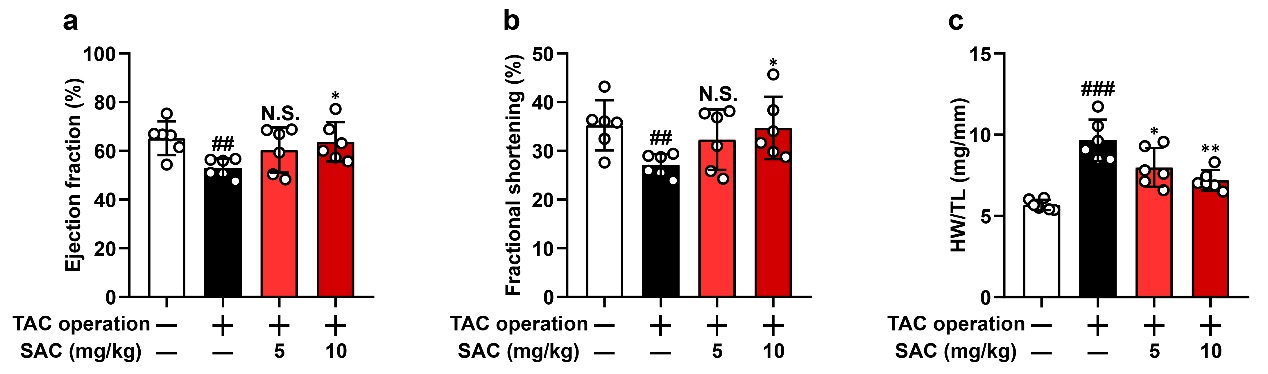


**Figure. S1 a-b** Echocardiographic analysis of left ventricular ejection fraction and fractional shortening in sham-operated, TAC-operated and SAC-treated mice (*n*=6). **c** The calculated heart weight (HW) to tibial length (TL) ratio in sham-operated, TAC-operated and SAC-treated mice (*n*=6). Data are represented as mean ± SD. ^##^*p* < 0.01*,* ^###^*p* < 0.001 *versus* control group. **p* < 0.05, ***p* < 0.01, N.S., nonsignificant *versus* model group.

**
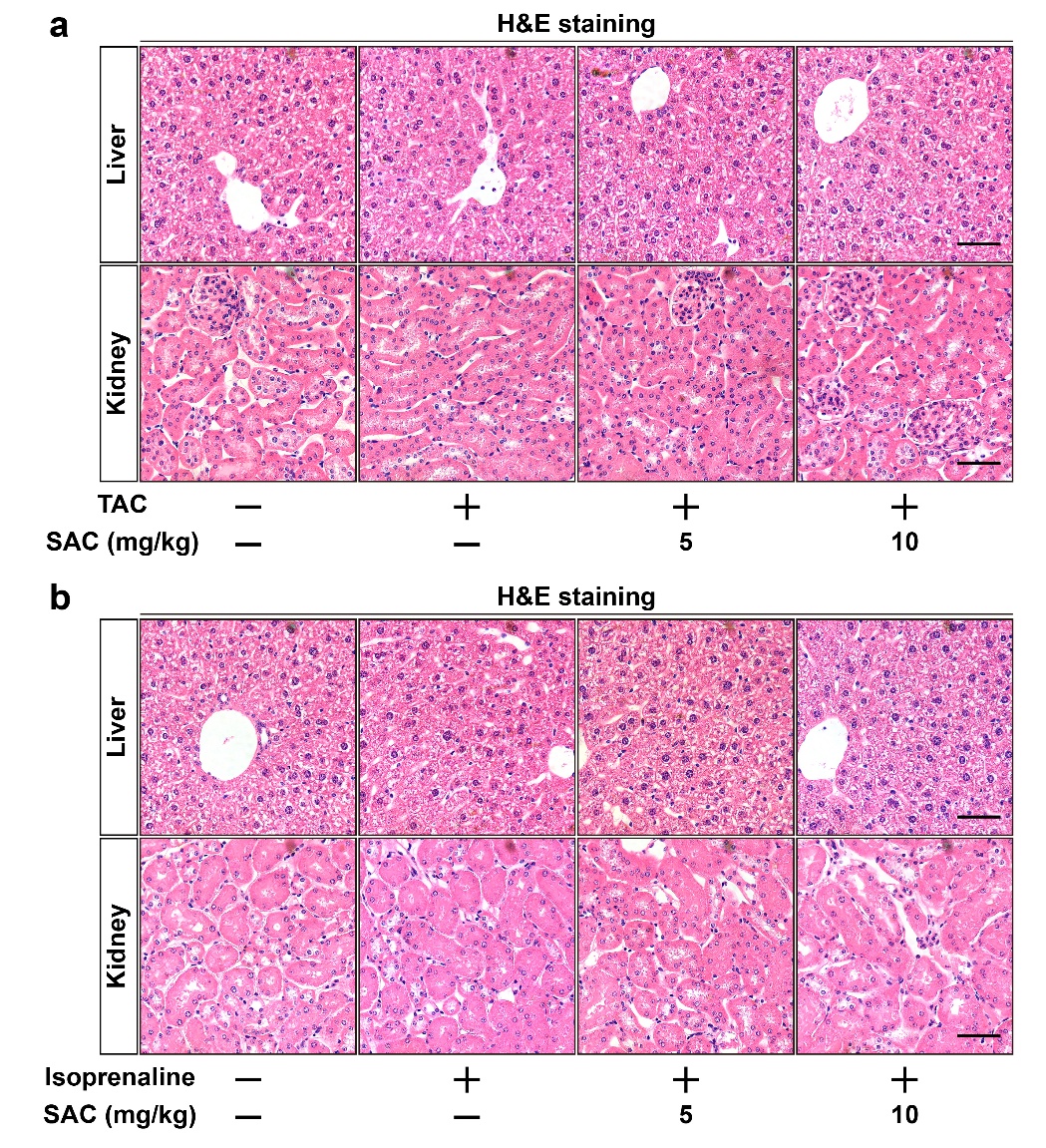
**

**Figure. S2** **a** Hematoxylin and Eosin (H&E) staining of liver and kidney sections from sham-operated, TAC-operated and SAC-treated mice, scale bar, 50 μm (*n*=6). **b** H&E staining of liver and kidney sections from vehicle, isoprenaline and SAC-treated mice, scale bar, 50 μm (*n*=6).

**
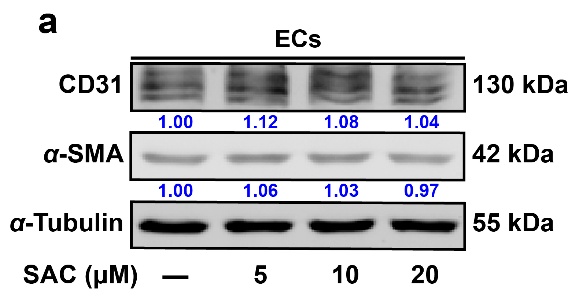
**

**Figure. S3** **a** Immunoblot analysis of CD31 and α-SMA expression in quiescent ECs treated with SAC in different concentrations. α-Tubulin was used as the loading control (*n*=3).

**
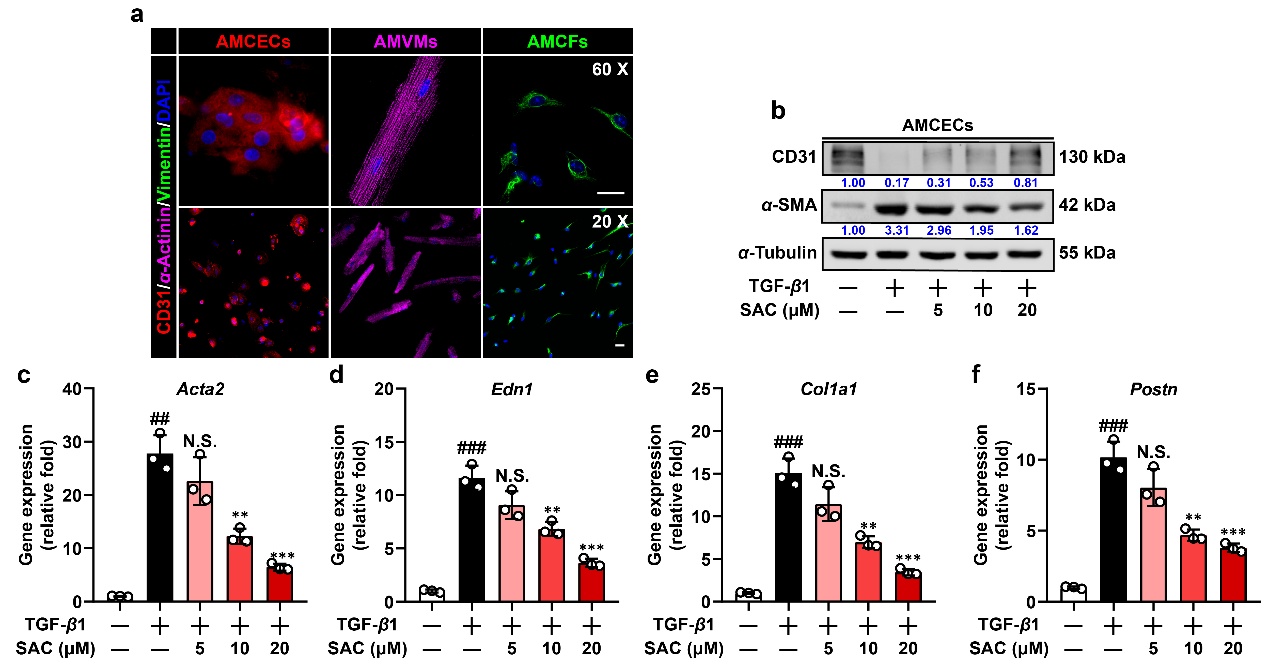
**

**Figure. S4 a** Immunofluorescent staining of endothelial marker CD31 (Red), cardiomyocyte marker α-Actinin (Purple) and fibroblast marker Vimentin (Green) in isolated adult mouse cardiac endothelial cells (AMCECs), ventricular myocytes (AMVMs) and cardiac fibroblasts (AMCFs), scale bar, 20 μm (*n*=3). **b** Immunoblot analysis of CD31 and α-SMA expression in AMCECs treated with vehicle, TGF-β1 and SAC. α-Tubulin was used as the loading control (*n*=3). **c-f** q-PCR analysis of *Acta2*, *Edn1*, *Col1a1* and *Postn* mRNA level in AMCECs treated with vehicle, TGF-β1 and SAC. *18s* RNA was used as the internal reference (*n*=3). Data are represented as mean ± SD. ^##^*p* < 0.01*,* ^###^*p* < 0.001 *versus* control group. ***p* < 0.01, ****p* < 0.001, N.S., nonsignificant *versus* model group.

**
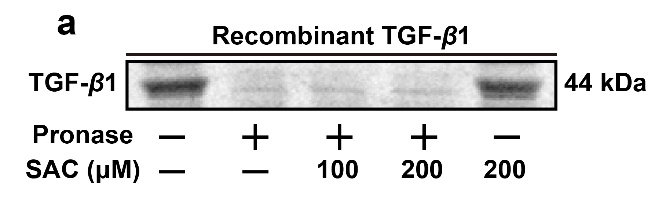
**

**Figure. S5** **a** Immunoblot analysis of TGF-β1 abundance of recombinant TGF-β1 protein following co-incubation with pronase or SAC, either alone or in combination at 37°C for 30 minutes (*n*=3).

**
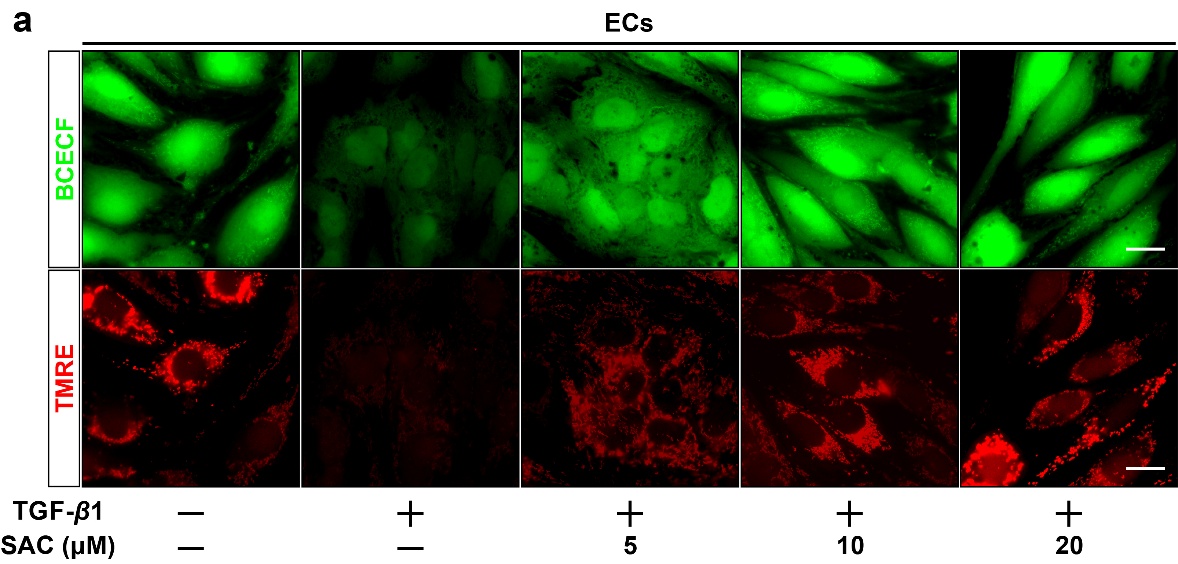
**

**Figure. S6** **a** Fluorescent counter-staining of TMRE and BCECF in ECs treated with vehicle, TGF-β1 and SAC, scale bar, 20 μm (*n*=3).

**
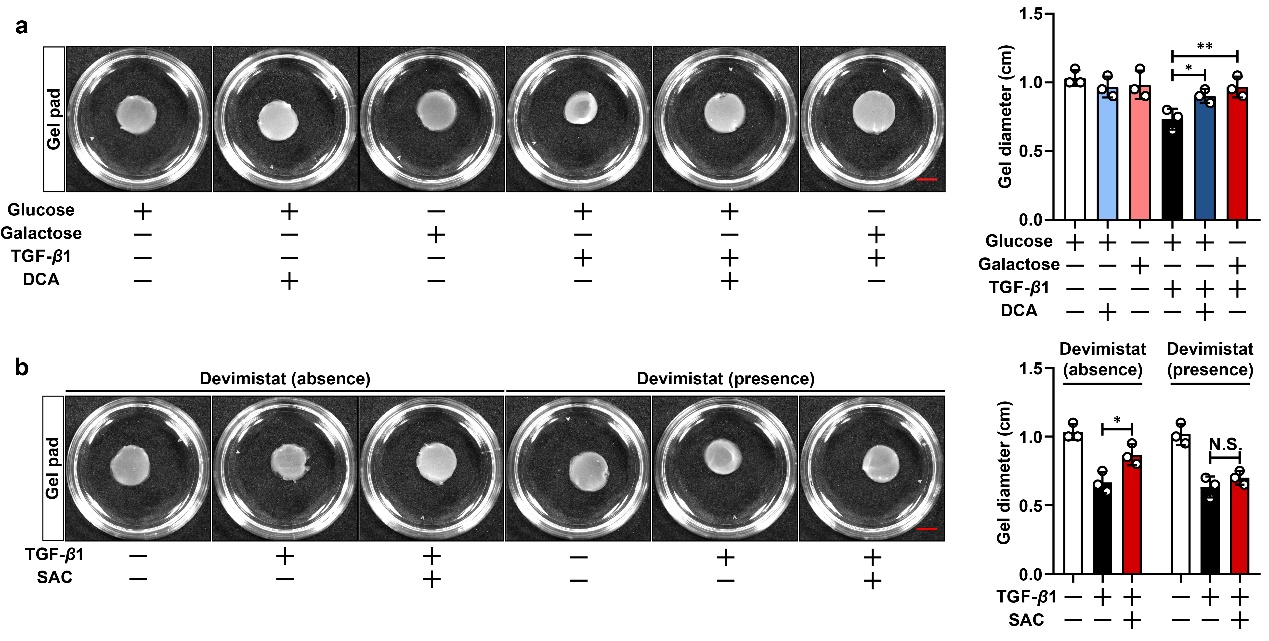
**

**Figure. S7** **a** Representative images and the calculated gel diameter in gel pad contraction assay of ECs in ECs treated with vehicle, TGF-β1, dichloroacetate (DCA) and galactose, scale bar, 5 mm (*n*=3). **b** Representative images and the calculated gel diameter in gel pad contraction assay of ECs in ECs treated with vehicle, TGF-β1, Salvianolic acid C (SAC) and devimistat, scale bar, 5 mm (*n*=3). Data are represented as mean ± SD. **p* < 0.05, ***p* < 0.01, N.S., nonsignificant *versus* indicated group.

**
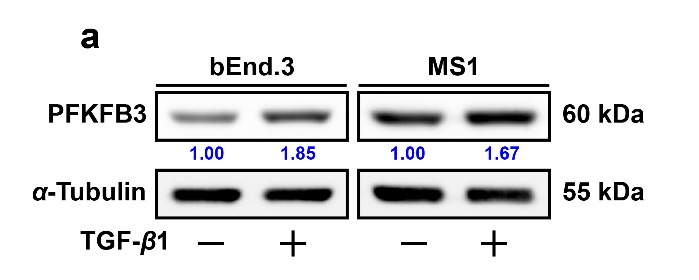
**

**Figure. S8** **a** Immunoblot analysis of PFKFB3 expression in bEnd.3 and MS1 cells treated with TGF-β1 for 96 hours. α-Tubulin was used as the loading control (*n*=3).

**
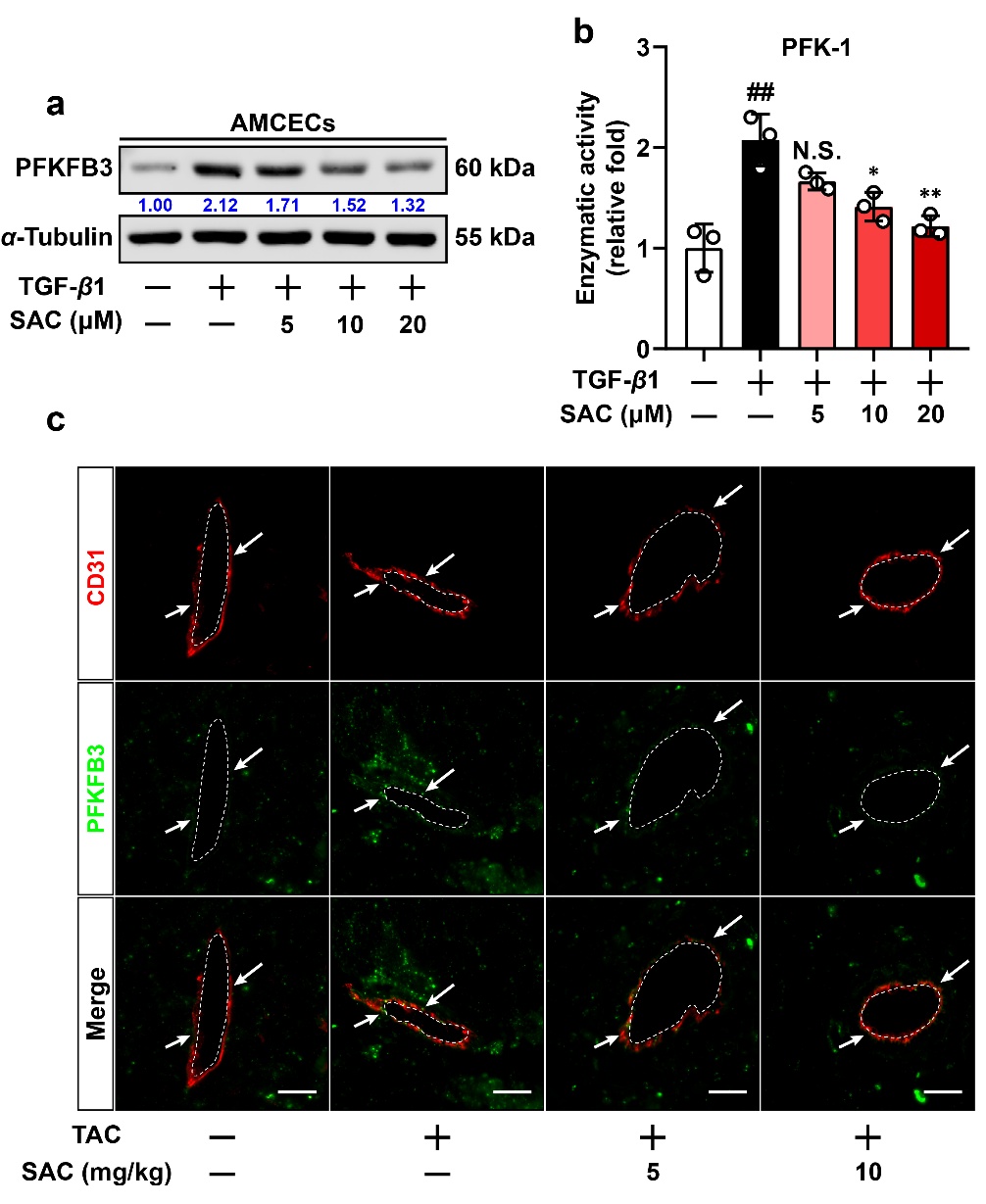
**

**Figure. S9** **a** Immunoblot analysis of PFKFB3 expression in AMCECs treated with vehicle, TGF-β1 and SAC. α-Tubulin was used as the loading control (*n*=3). **b** Enzymatic activity of PFK-1 in quiescent, transformed and SAC-treated AMCECs (*n*=3). **c** Immunofluorescent counter-staining of PFKFB3 (Green) and CD31 (Red) in cardiac sections (white dotted line was applied to delineate the inner surface of blood vessel) from sham-operated, TAC-operated and SAC-treated mice, scale bar, 20 μm. Data are represented as mean ± SD. ^##^*p* < 0.01 *versus* control group. **p* < 0.05, ***p* < 0.01, N.S., nonsignificant *versus* model group.

**
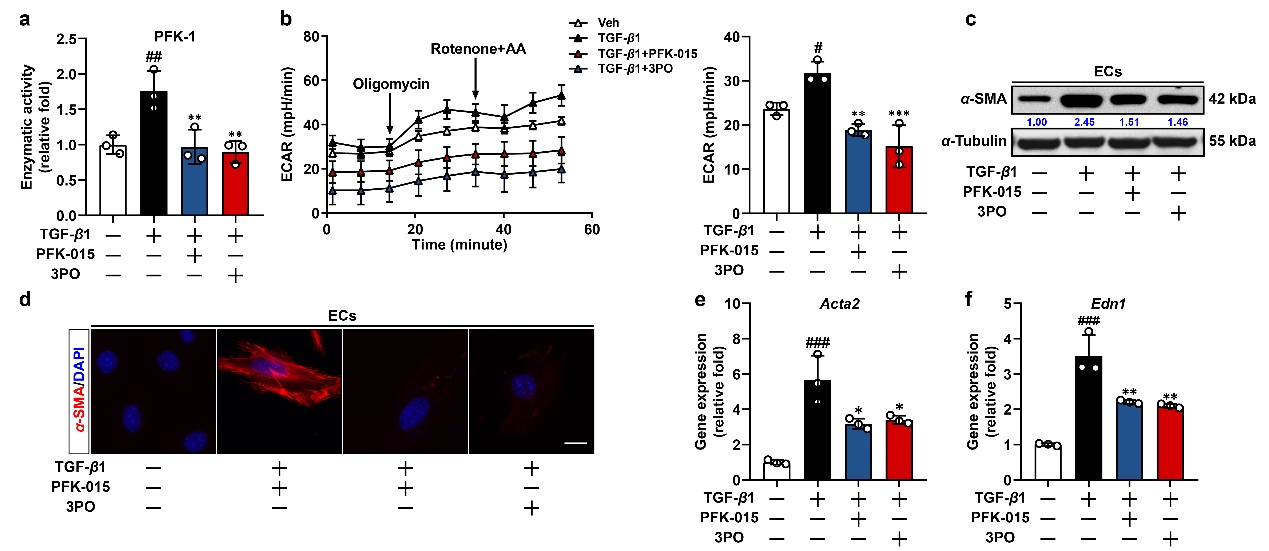
**

**Figure. S10** **a-b** Analysis of PFK-1 activity and ECAR in ECs treated with vehicle, TGF-β1, PFK-015 or 3PO (*n*=3). **c** Immunoblot analysis of CD31 and α-SMA expression in ECs treated with vehicle, TGF-β1, PFK-015 or 3PO. α-Tubulin was used as the loading control (*n*=3). **d** Immunofluorescent staining of α-SMA in ECs treated with vehicle, TGF-β1, PFK-015 or 3PO, scale bar, 20 μm (*n*=3). **e-f** q-PCR analysis of *Acta2*, and *Edn1* mRNA level in ECs treated with vehicle, TGF-β1, PFK-015 or 3PO. *18s* RNA was used as the internal reference (*n*=3). Data are represented as mean ± SD. ^#^*p* < 0.05, ^##^*p* < 0.01, ^###^*p* < 0.001 *versus* control group. **p* < 0.05, ***p* < 0.01, ****p* < 0.001 *versus* model group or indicated group.

**
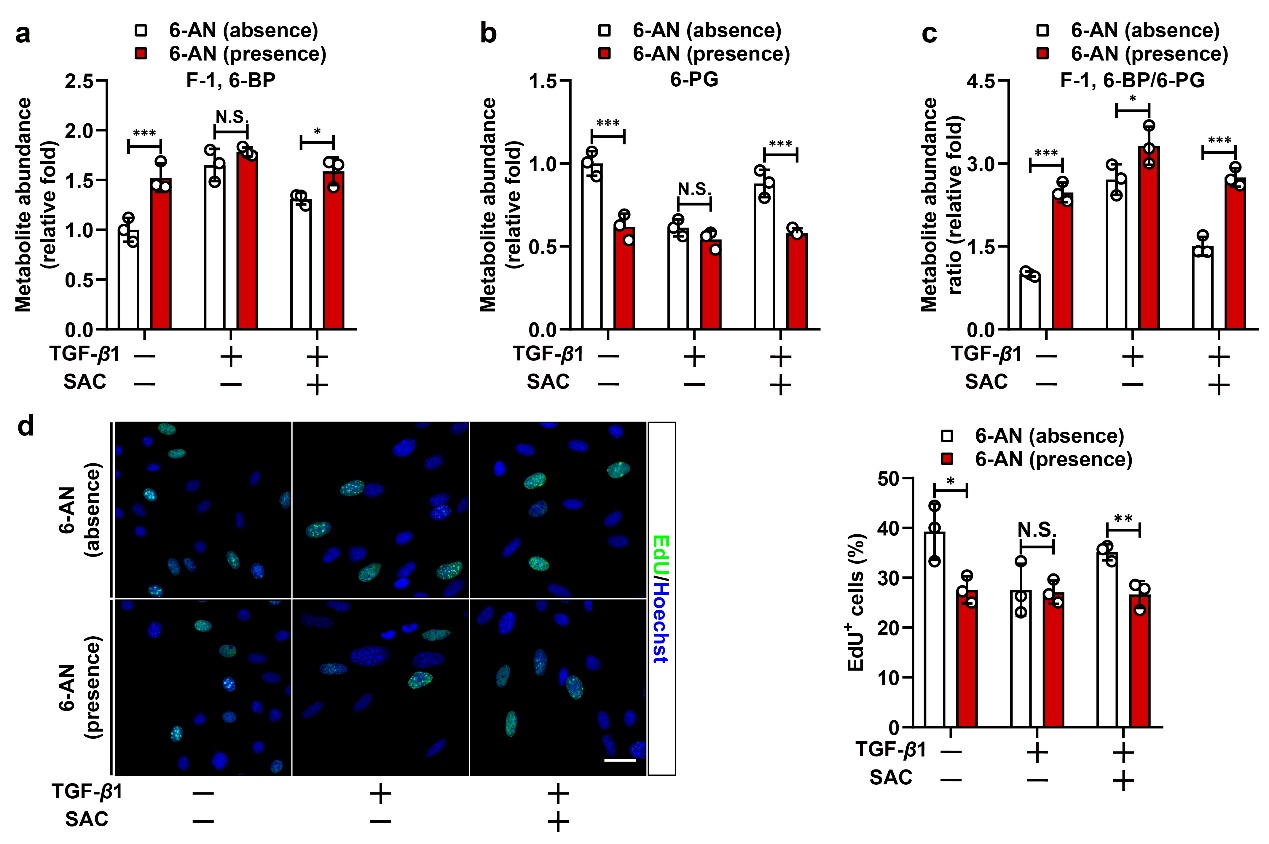
**

**Figure. S11** **a-c** Cellular F-1,6-BP, 6-PG abundance and F-1,6-BP/6-PG ratio in vehicle, TGF-β1, SAC-treated ECs in the absence or presence of 6-aminonicotinamide (6-AN) (*n*=3). **d** EdU incorporation assay of ECs and the calculated EdU-positive cell ratio in vehicle, TGF-β1, SAC-treated ECs in the absence or presence of 6-AN (*n*=3). Data are represented as mean ± SD. **p* < 0.05, ***p* < 0.01, ****p* < 0.001, N.S., nonsignificant *versus* indicated group.

**
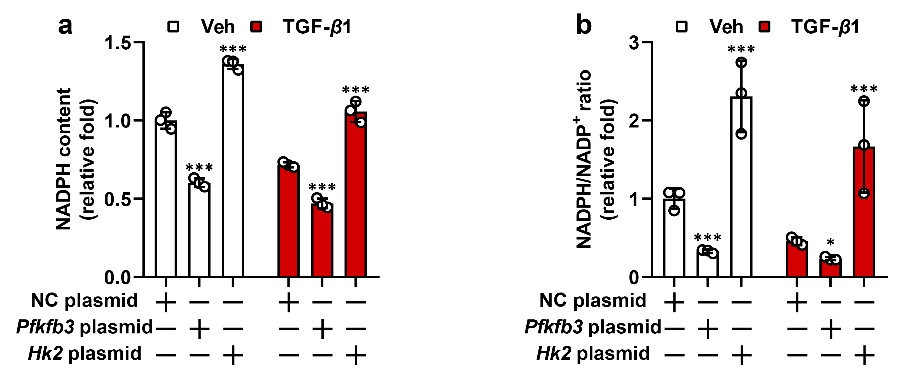
**

**Figure. S12** **a-b** Cellular NADPH abundance and NADPH/NADP^+^ ratio in negative control (NC), *Pfkfb3* or *Hk2* plasmid transfected ECs following vehicle and TGF-β1 treatment (*n*=3). Data are represented as mean ± SD. **p* < 0.05, ****p* < 0.001 *versus* negative control (NC) plasmid transfected cells.

**
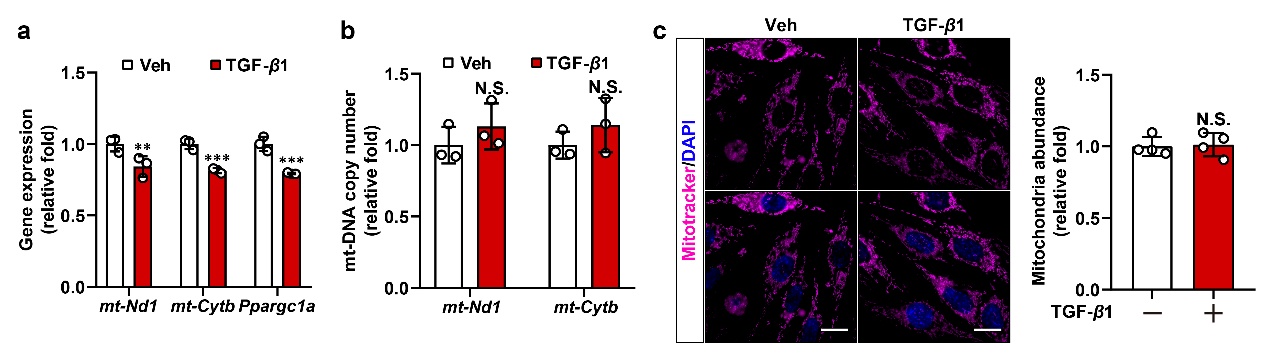
**

**Figure. S13** **a** q-PCR analysis of *mt-Nd1*, *mt-Cytb* and *Ppargc-1a* mRNA level in quiescent and transformed ECs. *18s* RNA was used as the internal reference (*n*=3). **b** q-PCR analysis of *mt-Nd1* and *mt-Cytb* mt-DNA copy number in quiescent and transformed ECs. A nuclear-encoded gene β*-actin* was used as the internal reference (*n*=3). **c** Fluorescent staining of Mitotracker and the calculated mitochondrial abundance in quiescent and transformed ECs, scale bar, 20 μm (*n*=4). Data are represented as mean ± SD. ***p* < 0.01, ****p* < 0.001, N.S., nonsignificant *versus* vehicle-treated ECs.

**
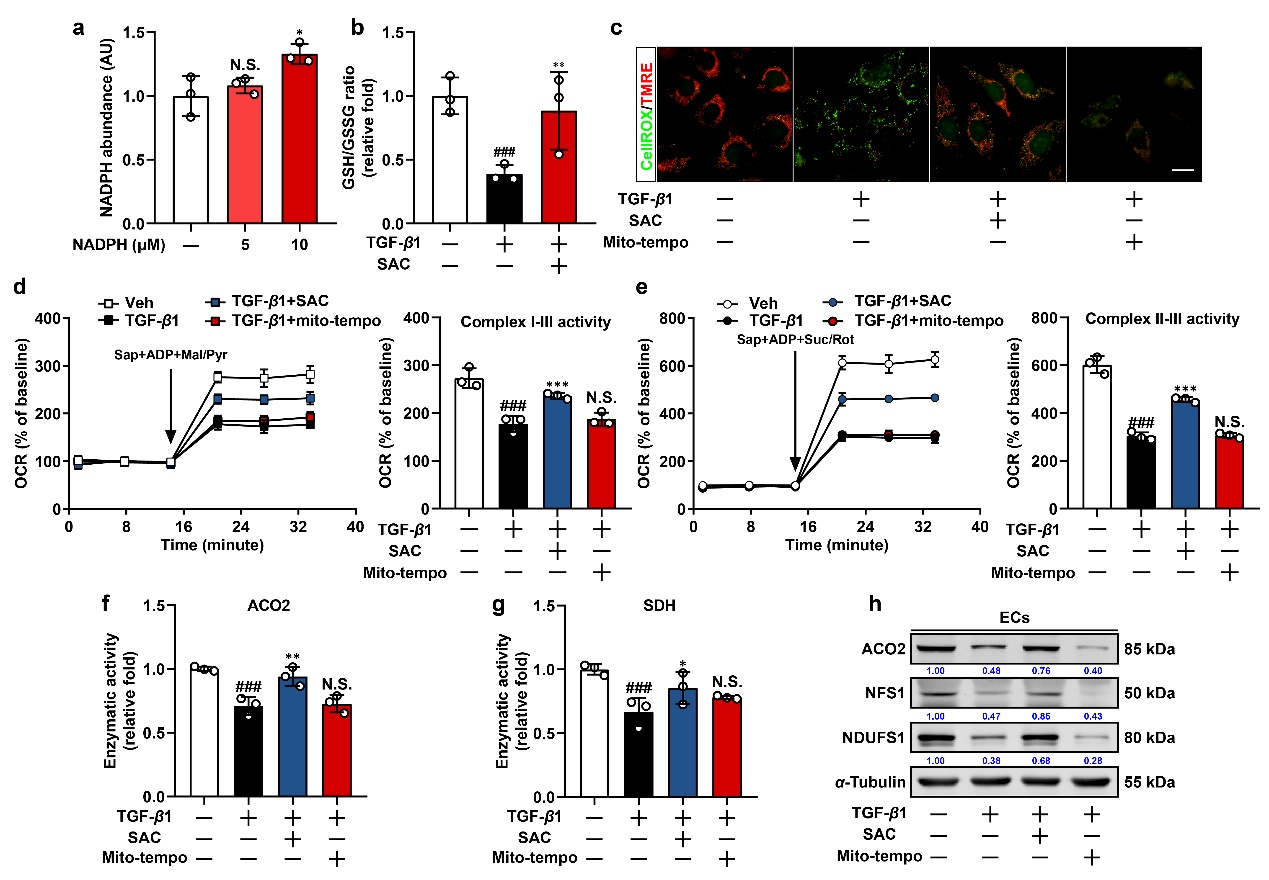
**

**Figure. S14** **a** Relative intracellular NADPH abundance post NADPH transfection (*n*=3). **b** GSH/GSSG ratio in quiescent, transformed and SAC-treated ECs (*n*=3). **c** Fluorescent counter-staining of CellROX and TMRE in quiescent, transformed, SAC-treated or mito-tempo-treated ECs, scale bar, 20 μm (*n*=3). **d-e** Average complex I–III and complex II-III activity in quiescent, transformed, SAC-treated or mito-tempo-treated ECs (*n*=3). **f-g** Average SDH and ACO2 activity in mitochondrial lysates of quiescent, transformed, SAC-treated or mito-tempo-treated ECs (*n*=3). **h** Immunoblot analysis of ACO2, NFS1 and NDUFS1 expression in quiescent, transformed, SAC-treated or mito-tempo-treated ECs. α-Tubulin was used as the loading control (*n*=3). Data are represented as mean ± SD. ^###^*p* < 0.001 *versus* control group. **p* < 0.05, ***p* < 0.01, ****p* < 0.001, N.S., nonsignificant *versus* control group (A) or model group (B-G).


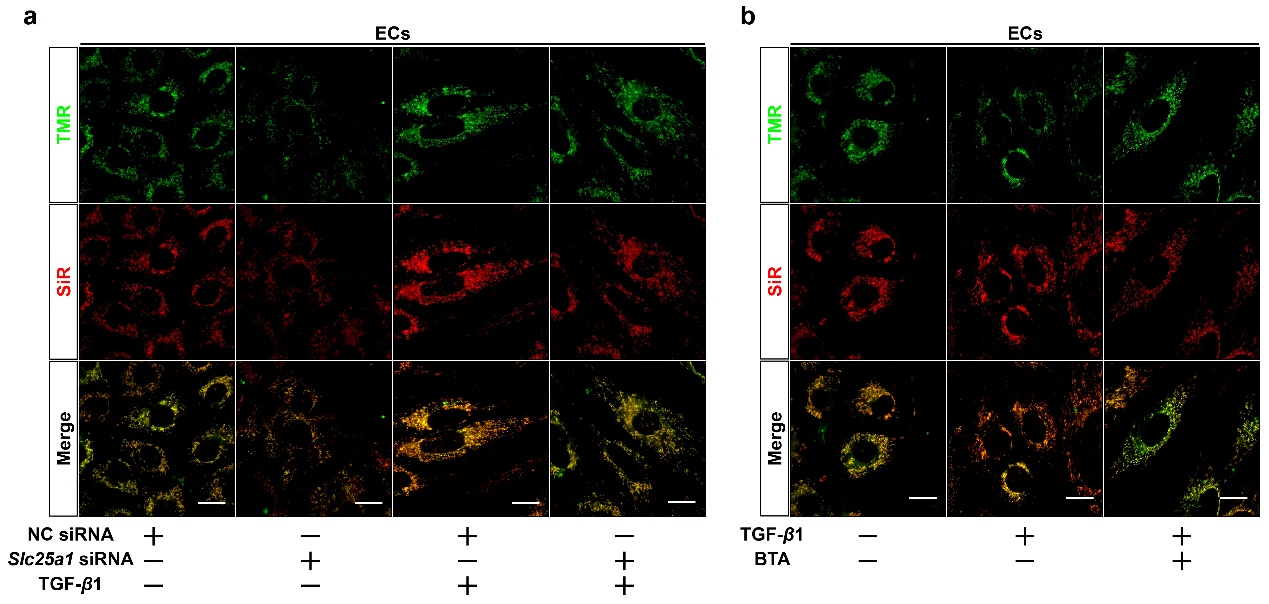


**Figure. S15** **a** Images of mitochondrial NADP-Snifit corresponding to the donor channel (TMR, green) and acceptor channel (SiR, red) in NC siRNA or *Slc25a1* siRNA transfected ECs post vehicle or TGF-β1 treatment, scale bar, 20 μm (*n*=3). **b** Images of mitochondrial NADP-Snifit corresponding to the donor channel (TMR, green) and acceptor channel (SiR, red) in quiescent, transformed and BTA-treated ECs, scale bar, 20 μm (*n*=3).


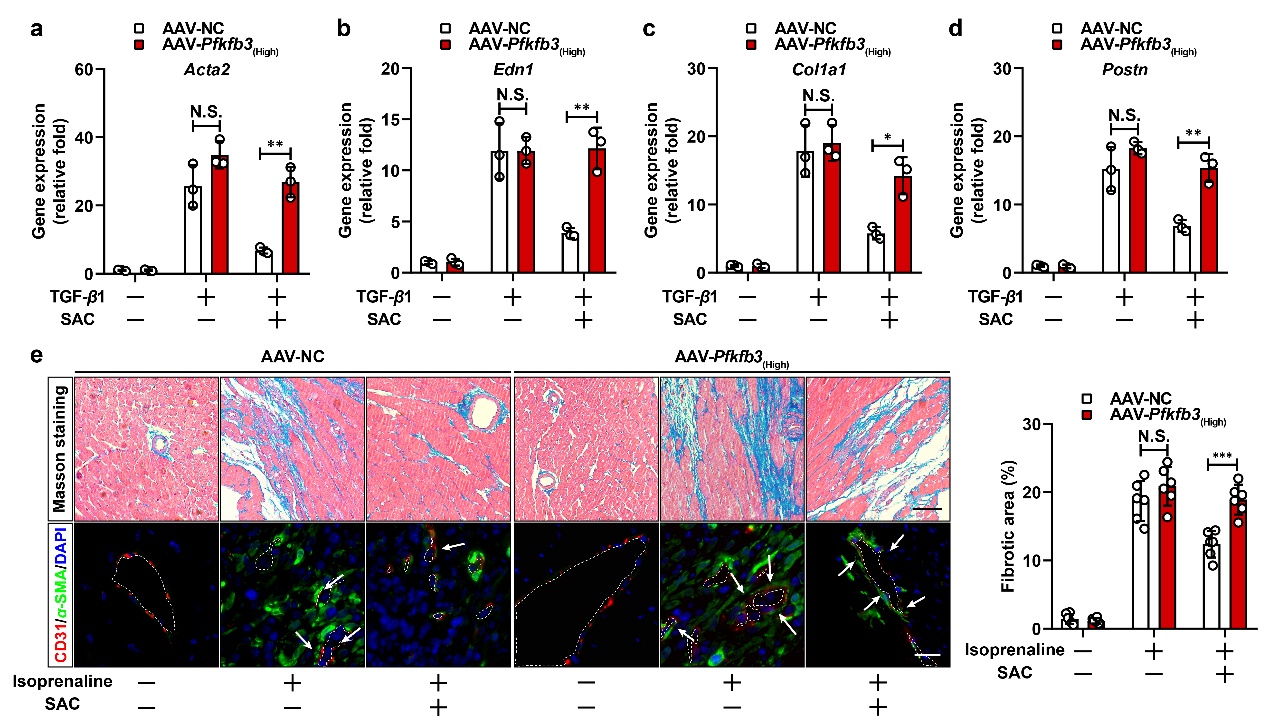


**Figure. S16** **a-d** q-PCR analysis of *Acta2*, *Edn1*, *Col1a1* and *Postn* mRNA level in AMCECs isolated from AAV-NC and AAV-*Pfkfb3*_(High)_ mice treated with vehicle, TGF-β1 and SAC. *18s* RNA was used as the internal reference (*n*=3). **e** Masson staining and immunofluorescent double-staining of CD31 (Red) and α-SMA (Green) of cardiac sections (white dotted line was applied to delineate the inner surface of blood vessel) from AAV-NC or AAV-*Pfkfb3*_(High)_ mice treated with vehicle, isoprenaline and SAC, scale bar, 50 μm (*n*=6). Data are represented as mean ± SD. **p* < 0.05, ***p* < 0.01, ****p* < 0.001 *versus* indicated group.

**
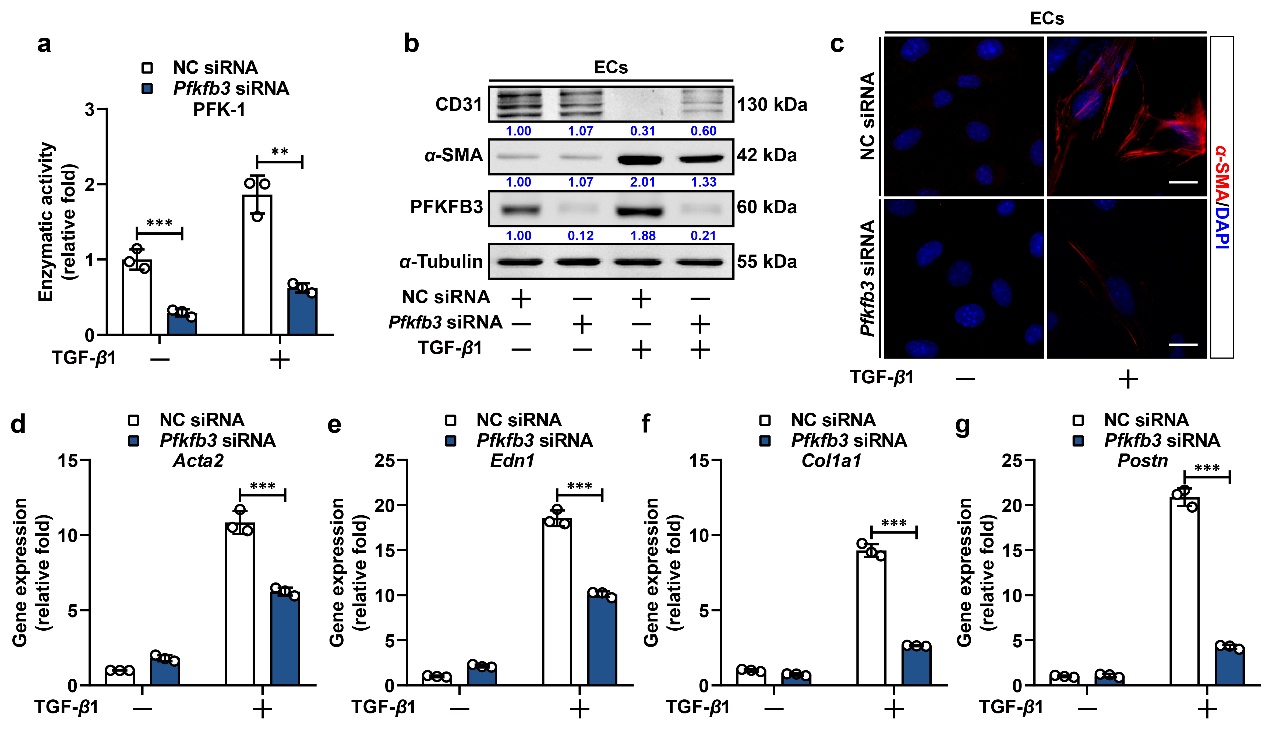
**

**Figure. S17** **a** PFK-1 activity in NC siRNA or *Pfkfb3* siRNA transfected ECs (*n*=3). **b** Immunoblot analysis of CD31, α-SMA and PFKFB3 protein expression in negative control (NC) or *Pfkfb3* siRNA transfected ECs post vehicle or TGF-β1 treatment. α-Tubulin was used as the loading control (*n*=3). **c** Immunofluorescent staining of α-SMA in negative control (NC) or *Pfkfb3* siRNA transfected ECs post vehicle or TGF-β1 treatment, scale bar, 20 μm (*n*=3). **d-g** q-PCR analysis of *Acta2*, *Edn1*, *Col1a1* and *Postn* mRNA level in negative control (NC) or *Pfkfb3* siRNA transfected ECs post vehicle or TGF-β1 treatment. *18s* RNA was used as the internal reference (*n*=3). Data are represented as mean ± SD. ***p* < 0.01, ****p* < 0.001 *versus* indicated group.

**
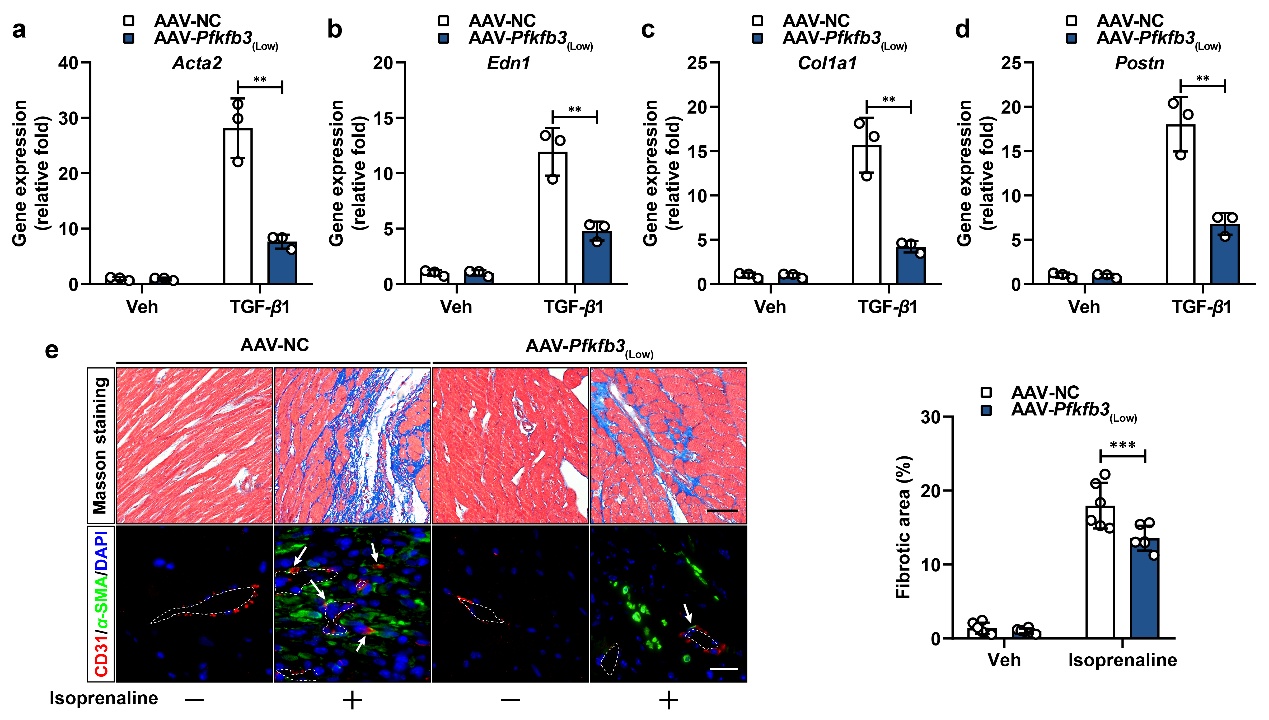
**

**Figure. S18 a-d** q-PCR analysis of *Acta2*, *Edn1*, *Col1a1* and *Postn* mRNA level in AMCECs isolated from AAV-NC and AAV-*Pfkfb3*_(Low)_ mice treated with vehicle, TGF-β1 and SAC. *18s* RNA was used as the internal reference (*n*=3). **e** Masson staining and immunofluorescent double-staining of CD31 (Red) and α-SMA (Green) of cardiac sections (white dotted line was applied to delineate the inner surface of blood vessel) from AAV-NC or AAV-*Pfkfb3*_(Low)_ mice treated with vehicle, isoprenaline and SAC, scale bar, 50 μm (*n*=6). Data are represented as mean ± SD. ***p* < 0.01, ****p* < 0.001, N.S., nonsignificant *versus* indicated group.

**Table S1 List of primary antibodies**

| ***Antibody*** | ***Catalog Number*** | ***Manufacturer*** |
| --- | --- | --- |
| *Anti-*α*-SMA antibody* | *ab7817* | *Abcam* |
| *Anti-PFKFB3 antibody* | *ab181861* | *Abcam* |
| *Anti-Sarcomeric* α*-Actinin antibody* | *ab68167* | *Abcam* |
| *Hexokinase I (C35C4) Rabbit mAb* | *2024* | *Cell Signaling Technology* |
| *Hexokinase II (C64G5) Rabbit mAb* | *2867* | *Cell Signaling Technology* |
| *PFKP (D4B2) Rabbit mAb* | *8164* | *Cell Signaling Technology* |
| *LDHA (C4B5) Rabbit mAb* | *3582* | *Cell Signaling Technology* |
| *GAPDH (D16H11) XP® Rabbit mAb* | *5174* | *Cell Signaling Technology* |
| *PKM1/2 (C103A3) Rabbit mAb* | *3190* | *Cell Signaling Technology* |
| *Vimentin (D21H3) XP® Rabbit mAb* | *5741* | *Cell Signaling Technology* |
| *CD31 Polyclonal antibody* | *11265-1-AP* | *Proteintech* |
| α*-Tubulin Monoclonal Antibody* | *66031-1-Ig* | *Proteintech* |
| *Aconitase 2 Polyclonal Antibody* | *11134-1-AP* | *Proteintech* |
| *NFS1 Polyclonal Antibody* | *15370-1-AP* | *Proteintech* |
| *NDUFS1 Polyclonal Antibody* | *12444-1-AP* | *Proteintech* |
| *NNT Polyclonal antibody* | *13442-2-AP* | *Proteintech* |
| *C5orf33 Polyclonal Antibody* | *26352-1-AP* | *Proteintech* |
| *IDH2 Polyclonal antibody* | *15932-1-AP* | *Proteintech* |
| *MTHFD2 Polyclonal Antibody* | *12270-1-AP* | *Proteintech* |
| *ME2 Monoclonal Antibody* | *67457-1-Ig* | *Proteintech* |
| *TGF Beta 1 Polyclonal Antibody* | *21898-1-AP* | *Proteintech* |
| *SLC25A1 Polyclonal antibody* | *15235-1-AP* | *Proteintech* |

**Table S2 Nucleotide sequences of siRNA**

| ***Names*** | ***Species*** | ***Sequence (5′ to 3′)*** |
| --- | --- | --- |
| *Pfkfb3* siRNA | *Homo sapiens* | GAAGAGGATCAGTTGCTATGATT |
| *Nfs1* siRNA | *Homo sapiens* | GAGATAGTATGTTAGAGTACATT |
| *Slc25a1* siRNA | *Homo sapiens* | GGCATTCTACAAGGGCACTGTTT |
| NC siRNA | *Homo sapiens* | GTAGGGTAGACGACATAGTTATT |

**Table S3 Nucleotide sequences of q-PCR primers**

| ***Names*** | ***Species*** | ***Sequence (5′ to 3′)*** |
| --- | --- | --- |
| *Acta2* | *Homo sapiens* | Sense: GCGTGGCTATTCCTTCGTTA  Antisense: ATGAAGGATGGCTGGAACAG |
| *Col1a1* | *Homo sapiens* | Sense: ATGTAGGCCACGCTGTTCTT  Antisense: GAGAGCATGACCGATGGATT |
| *Edn1* | *Homo sapiens* | Sense: TCAGAGGAACACCTAAGACAA  Antisense: TGCTCGGTTGTGGTCACATA |
| *Postn* | *Homo sapiens* | Sense: CCCCGTGACTGTCTATAAGCC  Antisense: TGACCTTGGTGACCTCTTCTTG |
| *Pfkfb3* | *Homo sapiens* | Sense: ACCAAAGATCACCCACGGATGT  Antisense: TGACCTTGGTGACCTCTTCTTG |
| *mt-Nd1* | *Homo sapiens* | Sense: CCCTAAAACCCGCCACATCT  Antisense: GAGCGATGGTGAGAGCTAAGGT |
| *mt-Cytb* | *Homo sapiens* | Sense: GGACTATTCCTAGCCATG  Antisense: CGGATGATTCAGCCATAA |
| *Ppargc1a* | *Homo sapiens* | Sense: TCTGAGTCTGTATGGAGTGACAT  Antisense: CCAAGTCGTTCACATCTAGTTCA |
| *Nnt* | *Homo sapiens* | Sense: GCTATGGTCTCTGTGCAGCC  Antisense: ATTAGCTAACAGCTTGATCC |
| *Nadk2* | *Homo sapiens* | Sense: CACTTAACCTCTGCCCGTCC  Antisense: TCGGTAGCAAGTCATCGTGG |
| *Mthfd2* | *Homo sapiens* | Sense: TACTCCATGGGGTGTGTGG  Antisense: GGGCATTCCAACGTTTT |
| *Idh2* | *Homo sapiens* | Sense: TCTGGCAGTTCATCAAGGAGAA  Antisense: GAGCCCGAGGTCAAAATACTTTAG |
| *Me2* | *Homo sapiens* | Sense: ATTAGTGACAGTGTTTTCCTA  Antisense: TCAGGTTCTGGGTATCG |
| β*-actin* | *Homo sapiens* | Sense: TGACGTGGACATCCGCAAAG  Antisense: CTGGAAGGTGGACAGCGAGG |
| *18s RNA* | *Homo sapiens* | Sense: TTTCGGAACTGAGGCCATGA  Antisense: GCAAATGCTTTCGCTCTGGTC |
| *Acta2* | *Mus musculus* | Sense: TGCTGGACTCTGGAGATGGTGTG  Antisense: CGGCAGTAGTCACGAAGGAATAGC |
| *Col1a1* | *Mus musculus* | Sense: GCTCCTCTTAGGGGCCACT  Antisense: CCACGTCTCACCATTGGGG |
| *Edn1* | *Mus musculus* | Sense: GCACCGGAGCTGAGAATGG  Antisense: GTGGCAGAAGTAGACACACTC |
| *Postn* | *Mus musculus* | Sense: CCTGCCCTTATATGCTCTGCT  Antisense: AAACATGGTCAATAGGCATCACT |
| *Pfkfb3* | *Mus musculus* | Sense: CAACTCCCCAACCGTGATTGT  Antisense: GAGGTAGCGAGTCAGCTTCTT |
| *18s RNA* | *Mus musculus* | Sense: CGGCTACCACATCCAAGGAA  Antisense: GCTGGAATTACCGCGGCT |
